# The G protein subunit α1, CaGα1, mediates ethylene sensing of mango anthracnose pathogen *Colletotrichum asianum* to regulate fungal development and virulence and mediates surface sensing for spore germination

**DOI:** 10.3389/fmicb.2022.1048447

**Published:** 2022-11-24

**Authors:** Chao-Yang Kao, Chun-Ta Wu, Hsien-Che Lin, Dai-Keng Hsieh, Huey-Ling Lin, Miin-Huey Lee

**Affiliations:** ^1^Department of Plant Pathology, National Chung Hsing University, Taichung, Taiwan; ^2^Advanced Plant Biotechnology Center, National Chung Hsing University, Taichung, Taiwan; ^3^Department of Horticulture and Landscape Architecture, National Taiwan University, Taipei, Taiwan; ^4^Department of Horticulture, National Chung Hsing University, Taichung, Taiwan

**Keywords:** mango anthracnose, *Colletotrichum asianum*, ethylene, self-inhibitors, G-protein, histidine kinase, signaling

## Abstract

Mango is an important tropic fruit, but its production is highly restricted by anthracnose diseases. Mango anthracnose development is related to the fruit-ripening hormone ethylene, but how the pathogen senses ethylene and affects the infection remains largely unknown. In this study, mango pathogen *Colletotrichum asianum* strain TYC-2 was shown to sense ethylene to enhance spore germination, appressorium formation and virulence. Upon further analysis of ethylene sensing signaling, three histidine kinase genes (*CaHKs*) and a G-protein gene (*CaGα1*) were functionally characterized. Ethylene upregulated the expression of the three *CaHKs* but had no influence on *CaGα1* expression. No function in ethylene sensing was identified for the three *CaHKs*. Ethylene enhanced spore germination and multiple appressorium formation of the wild-type TYC-2 but not CaGα1 mutants. TYC-2 has extremely low germination in water, where self-inhibition may play a role in ethylene sensing *via* CaGα1 signaling. Self-inhibitors extracted from TYC-2 inhibited spore germination of TYC-2 and CaGα1 mutants, but ethylene could not rescue the inhibition, indicating that the self-inhibition was not mediated by CaGα1 and had no interactions with ethylene. Interestingly, spore germination of CaGα1 mutants was significantly enhanced in water on hydrophobic but not hydrophilic surfaces, suggesting that CaGα1 is involved in surface sensing. In the pathogenicity assay, CaGα1 mutants showed less virulence with delayed germination and little appressorium formation at early infection on mango leaves and fruit. Transcriptome and qRT–PCR analyses identified several pathogenicity-related genes regulated by ethylene, indicating that ethylene may regulate TYC-2 virulence partially by regulating the expression of these genes.

## Introduction

Mango (*Mangifera indica* L.) is an important tropical fruit and is cultivated worldwide. However, its production is highly threatened by anthracnose disease caused by *Colletotrichum* spp. Anthracnose disease of mango displays dark-colored and necrotic lesions on foliage, flowers, and fruits. In particular, the pathogen remains quiescent on immature mango fruit but causes severe symptoms on ripe fruit, which results in severe losses at the postharvest stage (Arauz, 2000). *Colletotrichum* species spread through sexual and asexual spores. When a spore arrives at the host plant surface, the spore attaches, germinates, forms an appressorium and penetrates to build a parasitic relationship with the host, in which the appressorium is a specialized penetration structure generated by some fungal pathogens, including *Colletotrichum* species (Ryder and Talbot, 2015). Infection by *Colletotrichum* species can remain quiescent when the pathogen cannot receive signals released from the host, such as nutrients and the ripening hormone ethylene, and can overcome various stresses from the host or the environment, such as host defense compounds (Prusky, 1996; Arauz, 2000). Therefore, pathogen sensing signals from host plants are important for successful infection.

Host sensing is important for plant pathogens to establish infection, especially for spore germination and appressorium formation. Many host factors, including physical and chemical factors, can induce appressorium formation for initiating infection by plant pathogens; physical factors, such as surface topography, rigidity and hydrophobicity; and plant chemical components, such as ethylene, cutin and waxes (Kolattukudy et al., 1995; Kou and Naqvi, 2016). Among these factors, ethylene is very important in postharvest disease management on climacteric fruits. Interestingly, some plant pathogens can produce ethylene and/or sense ethylene, but the sensing and response to ethylene in fungi remain largely unclear. Ethylene is a signal for spore germination and/or invasion in *Botrytis cinerea* and some *Colletotrichum* species. In *B. cinerea*, low concentrations of ethylene induce spore germination and vegetative growth, while high concentrations of ethylene inhibit hyphal growth (Kępzyńska, 1989; Kępzyńska, 1993; Chagué et al., 2006). Ethylene can serve as a signal for spore germination and appressorium development in *C. gloeosporioides* and *C. musae* that infect the climacteric fruits avocado and banana, respectively (Flaishman and Kolattukudy, 1994).

In filamentous fungi, spore germination mechanisms and related signaling pathways have been well studied in *Aspergillus* spp. (Baltussen et al., 2019), while appressorium formation has been intensively studied in the rice blast pathogen *Magnaporthe oryzae* (Fernandez and Orth, 2018). The G-protein/cAMP pathway and mitogen-activated protein (MAP) kinase (MAPK) cascade are critical in *M. oryzae* appressorium formation for sensing physical surface cues (Fernandez and Orth, 2018). The G-protein, cyclic AMP, MAPK, or calcium/calmodulin pathway has been found in different *Colletotrichum* species involved in spore germination and/or appressorium formation (Kim et al., 1998, 2000; Truesdell et al., 2000; Barhoom and Sharon, 2004; Liu et al., 2018; Li et al., 2021).

Very few studies have been reported relating to signaling pathways involved in ethylene perception and signaling in fungi. The influences of ethylene on spore germination and the appressorium of *Colletotrichum* species were first demonstrated by Flaishman and Kolattukudy (1994) with ethephon treatment. Subsequently, the same research group showed that protein phosphorylation and a MAP kinase (CgMEK1) are involved in ethylene sensing in *C. gloeosporioides* (Kolattukudy et al., 1995; Kim et al., 2000). In *Botrytis cinerea* and *Verticillium dahlia,* fungal growth and/or virulence are affected by exogenous or endogenous ethylene. The two fungi are ethylene producers. Ethylene is proposed to be involved in symptom development caused by *V. dahliae*, in which the G protein β subunit and cAMP-dependent protein kinase A are involved in fungal ethylene production and virulence (Tzima et al., 2010, 2012). G-proteins are involved in the response of *B. cinerea* to ethylene. The growth of the class I Gα mutant is not strongly inhibited by ethylene as in the wild type, and ethylene biosynthesis is overproduced in the Gα mutant (Chagué et al., 2006).

In plants, ethylene sensing has much better characteristics than in fungi. Ethylene is perceived in plants by binding to histidine kinase (HK)-containing proteins, the ethylene-binding receptors (ETRs; Binder, 2008). Plant ETRs have multiple transmembrane domains at the N-terminus, a GAF domain at the center and an HK domain at the C-terminus. Upon binding with ethylene, the signal is transferred from the ETR to constitutive triple response 1 (CTR1) by repressing CTR1 activity and then activating a transcriptional cascade to regulate the expression of ethylene-responsive genes (Gallie, 2015). Five ETRs have been identified in *Arabidopsis thaliana* (Binder, 2008). Cynobacteria also have a similar ethylene sensing system (Wang et al., 2006; Lacey and Binder, 2016). Fungal HK proteins have more complicated functional domains, usually containing variable N-termini and conserved C-termini. The conserved C-terminus consists of three functional domains, histidine kinase A (HisKA), HK-like ATPase catalytic domain (HATPase) and receiver domain (RD; Hérivaux et al., 2016; Kabbara et al., 2019). HK proteins are involved in many responses in fungi to a wide range of environmental conditions (Defosse et al., 2015; Hérivaux et al., 2016). However, whether HK proteins have roles in ethylene sensing in fungi has not been documented.

To understand the influence of ethylene on the mango anthracnose pathogen *C. asianum*, potential signaling-related genes, including three HHK genes and the gene encoding the Gα1 protein, were disrupted in *C. asianum* strain TYC-2 for functional characterization. In addition, to identify TYC-2 genes responsive to ethylene, transcriptome and qRT–PCR analyses were conducted. Several ethylene-responsive genes are reported in this study.

## Materials and methods

### Fungal strains and cultivation

*Colletotrichum asianum* strain TYC-2 was isolated from diseased leaves of ‘Irwin’ mango in an orchard in Taiwan. Gene disruption mutants generated in this study were derived from the TYC-2 strain. All fungal strains were cultured on potato dextrose agar (PDA, Difco) medium and modified Mathur’s (MS) agar medium (Kuo et al., 2021) for regular growth and sporulation, respectively, under a 12-h light and 12-h dark cycle at 25°C for 6–8 days.

### Ethephon solution preparation and ethylene detection

Ethephon soluble concentrate (Yuan Mei, Taichung, Taiwan) was dissolved in water, and the pH was adjusted with 6 N NaOH to bring the stock solution to 500 μM at pH 8.0 immediately before conducting the experiment. Sealable 3.4-L plastic containers (Keyway, Miaoli, Taiwan) were used to generate ethylene from ethephon with a final volume of 50 ml. A tiny hole was created on the cover of the container and sealed with a piece of plastic tape to sample the air phase for ethylene detection. The concentration of ethylene was measured using gas chromatography (GC; GC-8A, Shimatsu, Japan), and 1 ppm ethylene was used as the standard to calculate the ethylene concentration.

### Ethephon treatment for spore germination

Multiple 10-μL spore suspensions (2×10^4^ spores/mL) were dropped on glass slides, plastic petri dishes, cover slips, water agar, or cellophane topping on 1.5% water agar or on glass slides and then immediately placed into sealable containers with ethephon treatments. For yeast extract pretreatment, a 10-μL spore suspension containing 5% yeast extract was dropped on a glass slide and incubated for 6 h. More than 100 spores were counted for germination percentage, and appressorium formation percentage was calculated based on germinated spores.

### Bioinformatic analysis of histidine kinase protein and G-protein α subunit

Genes encoding histidine kinase proteins and G-proteins were identified in the TYC2 genome (unpublished). The identified proteins were further analyzed with the Conserved Domains Database of NCBI, InterPro, and TMHMM Server v2.0. The gene accession numbers were listed in Supplementary Table 1.

### Plasmid construction and *Agrobacterium* T-DNA-mediated transformation

*Agrobacterium* T-DNA-mediated transformation (ATMT) combined with a split marker strategy was used to generate gene knockout mutants as described in Kuo et al. (2021). Approximately 1.5 kb of the 3′ flanking region and 5′ flanking region were amplified with primers with the designed restriction site *Xba*I or *Sac*I and cloned into the two binary vectors (p1300-3’-Hyg and p1300-5’-Hyg). The correct plasmids were transformed into *Agrobacterium tumefaciens* strain EHA105. By cocultivation of the two bacterial cells containing either p1300-3’hyg-5′-flanking or p1300-5’hyg-3′-flanking and TYC-2 spores, the two T-DNAs were transferred into the TYC-2 genome.

The gene complementation vector pN1300C-Gα1 was constructed by cloning the PCR product of the CaGα1 gene with 1.5-kb up- and 0.3-kb downstream regions into the binary vector pN1300C (Lee et al., 2010) and the vector was transferred by ATMT into the mutant strain Gα1-23 as described above. All primers used are listed in Supplementary Table 2.

### DNA extraction, PCR screening and Southern blotting

Genomic DNA was extracted from mycelia cultured on PDA plates topped with a layer of cellophane and extracted using the hexadecyltrimethyl ammonium bromide (CTAB) extraction method (Hsieh et al., 2022). The DNA pellet was resuspended in 30–50 μl sterile Milli-Q water with 20 ng/μL RNase A and used in PCR screening and Southern blotting. PCR assays were used to screen transformants carrying three DNA homologous recombination events, including the crossover of two split markers, the crossover of the 5′ flanking region of T-DNA and the endogenous locus, and the crossover of the 3′ flanking region of T-DNA and the endogenous locus. Southern blot analysis was performed as described previously (Kuo et al., 2021).

### RNA-seq analysis

TYC2 was grown by plating out 200 μl spore suspension (1×10^5^ spores/mL) on PDA plates covered with cellophane for 2 days. The PDA plates were then incubated with different concentrations of ethephon solution in a 3.4-L sealed container for 3 h. To verify that these ethephon treatments, a petri dish plate with spore suspension drops was placed in each container. Spore germination and appressorium formation were measured to ensure that the treatments worked well.

For RNA extraction, mycelia were harvested from three replicates for each treatment, and total RNA was extracted with TRIzol (Invitrogen, USA) according to the instructions of the manufacturer. Two sets of RNA from two independent experiments were used for RNA-seq and comparative transcriptomic analysis, which were performed by a local company (Genomics BioSci & Tech Co Ltd., Taiwan). Briefly, the qualified RNA was used for library generation using the TruSeq Stranded mRNA Library Prep Kit (cat# RS-122-2,101, Illumina, San Diego, CA, USA) following the manufacturer’s recommendations, and index codes were added to attribute sequences to each sample. The final library quality was assessed on the Agilent Bioanalyzer 2,100 system using DNA High Sensitivity Chips. The libraries were sequenced on an Illumina NovaSeq 6000 platform, and 150 bp paired-end reads were generated. After sequencing, low-quality bases (<Q20) and adapters were removed by Trimmomatic v0.36.

For comparative transcriptomic analysis, the paired-end reads were mapped to the TYC-2 CDS by “Bowtie2 v2.3.4.3,” which was generated from the TYC-2 genome using the MAKER gene annotation pipeline. The gene expression count was calculated using “RSEM v1.2.28” after combining the reads from two independent experiments in each treatment. Differentially expressed genes were calculated using “egdeR v3.24.1.” Functional annotation was performed for all transcripts, including BLAST, COG, GO, Pfam database, and KEGG analyses.

### Relative gene expression by qRT–PCR assay

RNA was extracted as described above. Complementary DNA was synthesized by using M-MLV reverse transcriptase (M-MLV RT; Invitrogen, U.S.A.) and oligo (dT) as the primer at 37°C for 1 h. Gene expression levels were detected with qRT–PCR with a Rotor-Gene Q PCR machine (QIAGEN, Valencia, CA, USA). Primers (Supplementary Table 1) were designed using NCBI primer-blast.^1^ PCRs were performed with 5× HOT FIREPol EvaGreen qPCR Mix Plus (Solis BioDyne) using the program: 10 min at 95°C, followed by 40 cycles of 94°C for 15 s, 60°C for 20 s and 72°C for 20 s. The relative expression levels were detected using the comparative ΔΔCT method as described previously (Yu et al., 2017). The actin gene (*CgAct*) of TYC-2 was used as an internal control.

### Pathogenicity assay

Inoculation experiments were performed with 3- to 10-day-old detached young leaves of ‘Irwin’ mango grown in a greenhouse and fruits of ‘Irwin’ mango purchased from local markets. Divided by the central vein of a leaf, TYC-2 was inoculated on the left side, and a transgenic strain was inoculated on the right side of the same leaf. For fruit inoculation, different fungal strains were inoculated in parallel on a fruit surface. Wound inoculation was conducted by punching the infection site with a sterile pin immediately before inoculation. A 5-μL spore suspension (1×10^5^ spores/mL) was dropped on the plant surface, with multiple drops per leaf/fruit. Lesion sizes were measured by using ImageJ.

### Fungal growth and sporulation assay

Fungal growth assays were performed on three different agar media including PDA, MS and modified Czapek-Dox medium (Kuo et al., 2021). A 5-μL spore suspension (1×10^5^ spores/mL) was placed on the center of the medium plate for 6 days. The colony size was calculated using ImageJ, and spores were collected and counted from MS agar medium. Sporulation was presented based on colony size (cm^2^).

### Self-inhibitor extraction and analysis

TYC-2 grown on MS agar medium for 9 days was used for self-inhibitor extraction. The extraction and thin-layer chromatography (TLC) assay were performed by following the descriptions in Tsurushima et al. (1995) and Yu et al. (2020) with modifications. TYC-2 spores were collected into a sterile tube with 1 ml ethyl acetate (EA) after the cultures had been dried in a fume hood for 30 min, and the tube was placed at 4°C for 16 h. Spores were removed and EA extract was used in a TLC assay.

TLC assays were performed with a silica gel plate (60 F_254_, Merck, Germany) with the developing solvents hexane, EA and methanol (79:20:1; vol/vol/vol) and examined under UV (254 nm) or near-UV light (365 nm) after development. The developed TLC plate was topped on 1% water agar and then covered with 0.7% PDA containing TYC-2 spores (2×10^5^ spores/mL) and incubated for 2 days. The mycelia were stained with lactophenol cotton blue solution (Yu et al., 2020). The fractions with fungal growth inhibition were removed from TLC and dissolved in ethanol for spore germination assays on petri dishes.

### Analysis of experimental data

Experiments were conducted at least twice with at least three replicates of each treatment within each experiment, except as noted otherwise. The significance of differences was determined with a paired *t-test* or Tukey’s HSD test at *p* < 0.05 using Statistical Package for the Social Sciences software, version 20 (IBM SPSS software).

## Results

### Ethephon enhanced spore germination, appressorium formation and virulence in *Colletotrichum asianum* strain TYC-2

**Figure 1:**
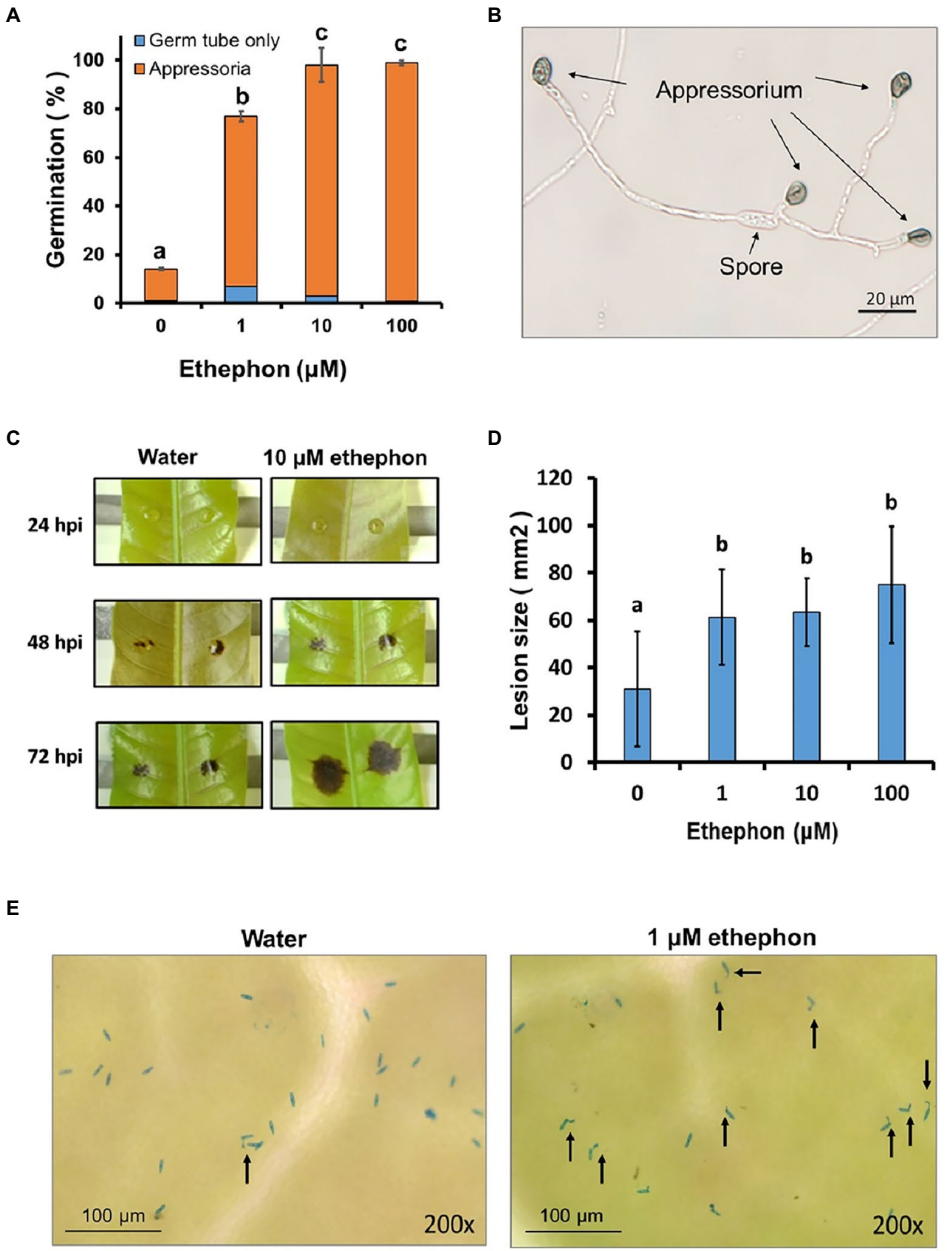
The effect of ethephon treatments on spore germination, appressorium formation **(A,B)** and virulence **(C–E)** of *Colletotrichum asianum* TYC-2. **(A)** Spore germination and appressorium formation of TYC-2 on glass slides at 24 h under 0–100 μM ethephon treatments. **(B)** Multiple appressorium formation under 100 μM ethephon treatment. **(C)** Lesion sizes caused by TYC-2 on young mango leaves under water and 10 μM ethephon treatment at 24–72 h postinoculation (hpi). **(D)** Lesion size at 72 hpi was measured with ImageJ. **(E)** Spore germination and appressorium formation on the leaf surface at 2 hpi. The data were analyzed by one-way ANOVA, and different letters indicate significant differences between treatments (*p* value <0.05) on germination **(A)** and lesion size **(B)**.

Ethylene can be released from ethephon solution, which is commonly used in postharvest treatments. To understand the roles of ethylene in the fungal development and virulence of mango anthracnose pathogen *C. asianum* strain TYC-2, the ethylene generator ethephon was used in the assays. At 24 h postincubation (hpi), 1 and 10 μM ethephon increased spore germination from less than 15% to over 75%. Over 90% of the germinated spores formed appressoria in all treatments, but multiple appressoria were significantly found in 10 and 100 μM ethephon treatments (Figures 1A,B). In the inoculation assay on mango leaves, tiny necrotic lesions appeared at 24 hpi and gradually enlarged at 48–72 hpi. Larger lesion sizes were found in the ethephon treatments than in the water treatment at 72 hpi (Figures 1C,D). Most of the spores germinated on the leaf surface under 1 μM ethephon treatment, but very few spores germinated in the water treatment at 2 hpi (Figure 1E). It suggests that the lesion size increased by ethylene might be mainly due to the increase of spore germination and appressorium formation.

The effects of ethylene on the growth and sporulation of TYC-2 were assayed on PDA and MS agar media. The results showed no significant difference among the 0–100 μM ethephon treatments on colony size and sporulation 4 days postinoculation (dpi) (Supplementary Figure 1).

### Ethylene released from ethephon solution in the assay system was concentration- and incubation time-dependent

To understand the true amount of ethylene released and the release patterns in our assay system, the ethylene concentration was detected. The concentrations of ethylene were gradually increased from 1 or 10 μM ethephon solutions by increasing the incubation time. The conversion of ethylene from ethephon displayed a linear reaction within 6 h of incubation and then slowed down and saturated at 24 h. In the 1 μM ethephon, approximately 1 ppm and 2 ppm ethylene were detected at 3 and 6 h, respectively, and the concentration of ethylene reached approximately 3.5 ppm at 26 h. Ethylene amounts in the containers with 10 μM ethephon were approximately 10 times that of ethylene in 1 μM ethephon containers at every incubation time point. Approximately 10 ppm and 20 ppm ethylene were converted from 10 μM ethephon after incubation for 3 h and 6 h, respectively (Supplementary Figure 2A).

### Ethylene induced spore germination and appressorium formation of TYC-2 after a short incubation time

To determine how much incubation time is required for ethylene to induce TYC-2 germination, TYC2 spore germination under different concentrations of ethephon treatments (0–100 μM) at 2, 3, and 6 h was detected. No linear germination pattern related to the incubation times was found in the 10 and 100 μM ethephon treatments for 2 to 6 h (Supplementary Figure 2B). However, a linear pattern of germination percentage to incubation time was found in the 1 μM ethephon treatment. The data revealed that TYC2 spore germination occurred in an incubation time-dependent manner under 1 μM ethephon treatment (Supplementary Figure 2B). Germination could reach over 50% under 1 μM ethephon treatment at 3 h and appressorium formation was enhanced by 1–100 μM ethephon treatments compared to the control (0 μM ethephon) at 2 h (Supplementary Figure 2B). Appressorium formed immediately after spore germination (Supplementary Figure 2C). To quickly investigate genes involved in ethylene sensing, the treatment with ethephon solution for 3 h was used for functional analysis of the selected genes in response to ethylene on spore germination and appressorium formation as described below.

The overall TYC-2 spore germination process under 10 μM ethephon treatment for 6 h is shown in Supplementary Figure 2C. Spore germination was observed at 0.5 h, and a swollen germ tube for appressorium formation initiation was formed at 1 h. The melaninization of the appressorium cell wall was observed at 6 h. This germination and appressorium formation process could also be observed in the 1 μM ethephon and water treatment, but the process was much slower, with very few germinated spores under the water treatment.

### The expression of three HK genes was slightly upregulated by ethylene

**Figure 2:**
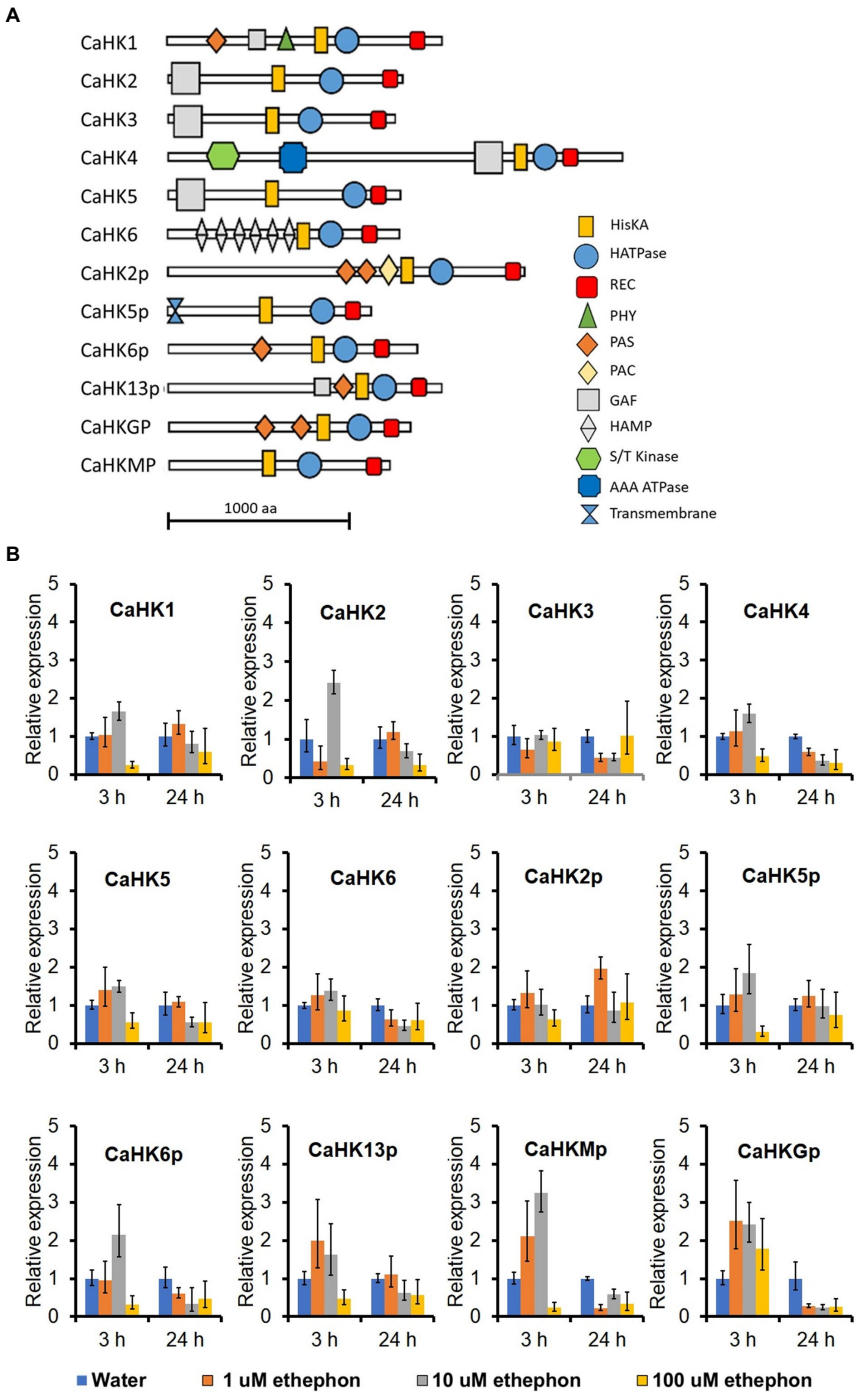
The protein structure and functional domain of the 12 histidine kinase (HK) genes of *Collototrichum asianum* TYC-2 **(A)** and their relative expression levels at 3 and 24 h under 0, 1, 10 and 100 μM ethephon treatments **(B)**. Relative expression was determined using actin gene as internal control and calculated with comparative ΔΔCt method by comparing to water treatment at each time point.

Twelve HK genes were identified in the TYC-2 genome, encoding proteins with approximately 1,200–2,500 amino acids (Figure 2A). Multiple functional domains were predicted in the 12 proteins. Three domains existed in the 12 proteins, including histidine kinase (HisKA), histidine-like ATPase (HATPase), and signal receiver (Rec), with the order from the N-terminus to the C-terminus. Additional varied domains located at the N-terminus were found in several of the HK proteins (Figure 2A). Gene expression of the 12 HKs was analyzed in TYC-2 mycelia at 3 and 24 h after treatment with 0–100 μM ethephon (Figure 2B). Three genes showed slightly higher expression levels than water treatment at 3 h after ethephon treatments, including *CaHK2*, *CaHKMp* and *CaHKGp*. *CaHK2* was upregulated under 10 μM ethephon treatment. *CaHKMp* was upregulated under 1 and 10 μM ethephon treatments, while *CaHKGp* was upregulated under 1–100 μM ethephon treatments. However, no upregulation of the 12 genes was found at 24 h.

To understand the function of the three genes upregulated by ethylene in TYC-2, gene disruption mutants were generated by replacing the histidine kinase domain with the hygromycin resistance gene (*hptII*) cassette. The potential transformants were screened with PCR for three DNA crossover events. Further verification for a single specific insertion was performed with Southern blotting (Supplementary Figure 3). Two to three gene-disrupted mutants without nonspecific insertions were obtained for each gene and used for further analysis of ethylene sensing for spore germination.

### The three HK genes were not involved in ethylene sensing for spore germination and appressorium formation in TYC-2

The three CaHK gene mutants had extremely low germination ability in the water treatment, but germination was largely increased in the 1 and 10 μM ethephon treatments. However, no significant differences were detected between the mutants and the wild-type strain TYC-2. For appressorium formation, extremely low germination resulted in a highly varied appressorium formation percentage in the water treatment, but no significant difference between the mutants and TYC-2 was found in the ethephon treatments (Supplementary Figure 4).

### *CaGα1* encoding the G-protein α1 subunit was not regulated by ethylene

CaGα1 was identified in the genome of TYC-2 as a homolog of class I Gα protein (Gα1). CaGα1 encodes 353 amino acids, and the Gα domain (61–181 a.a.) was predicted (Supplementary Figure 5). The expression level of CaGα1 under ethylene treatment was analyzed. No significant influence on the expression level of CaGα1 was detected under 1 and 10 μM ethephon treatments (Supplementary Figure 5).

**Figure 3:**
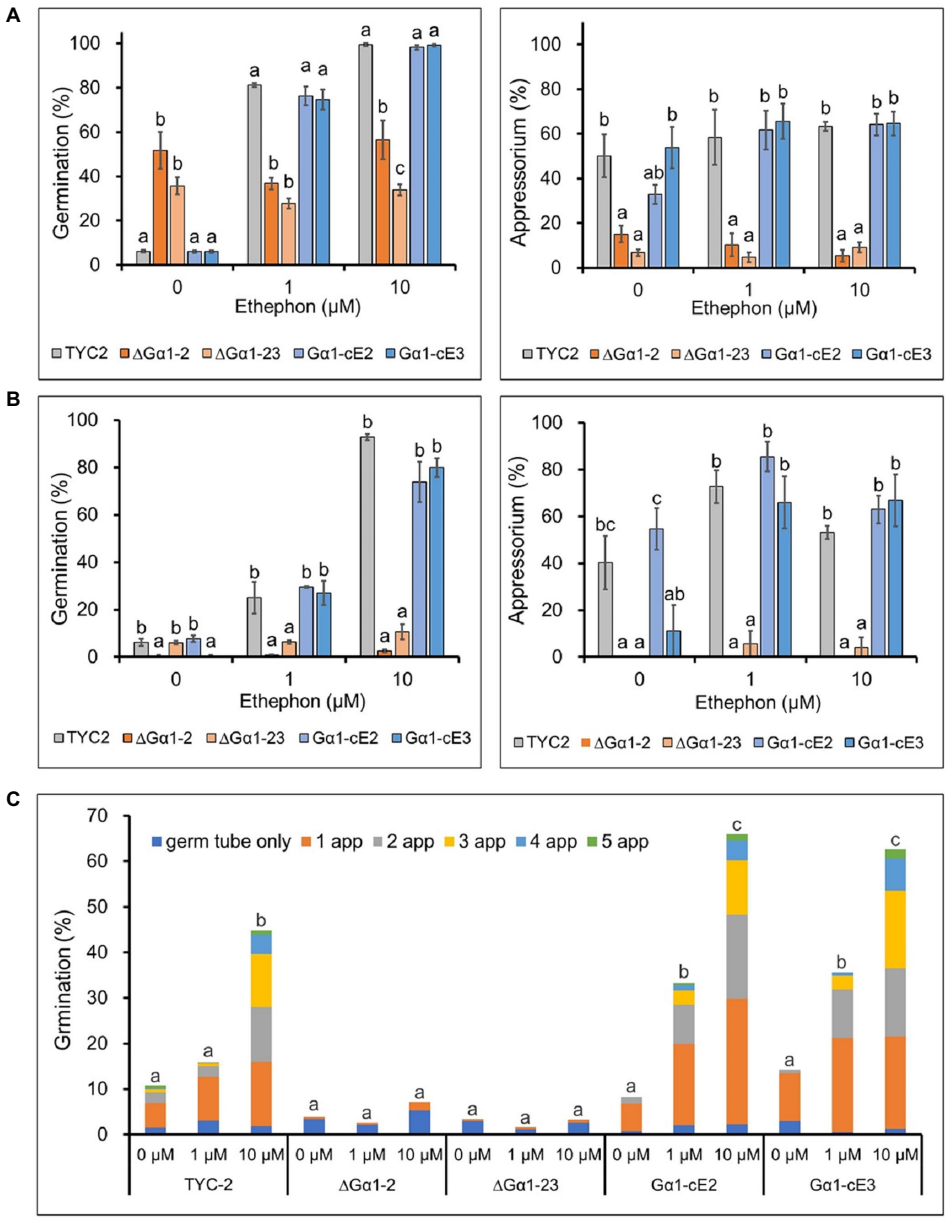
Spore germination and appressorium formation of *Colletotrichum asianum* wild-type strain (TYC-2), G𝛼1 mutants (∆G𝛼1–2, ∆G𝛼1–23) and gene complementation strains (G𝛼1-cE2, G𝛼1-cE3) on plastic petri dishes **(A)** and glass slides **(B)** at 3 h under 0–10 μM ethephon treatments. The data within same treatment were analyzed by one-way ANOVA, different letters indicate significant differences between treatments (*p* value <0.05). Multiple appressorium formation on glass slides at 48 h under 0–10 μM ethephon treatments **(C)**. The multiple appressorium formation (>2 appressoria) percentage of all treatments was used for statistic analysis by one-way ANOVA, different letters indicate significant differences between treatments (*p* value <0.05).

The *CaGα1*-disrupted mutants in *C. ansianum* TYC-2 were analyzed with PCR and Southern blotting. Hybridization of the *Sal*II-digested genomic DNA with the *hptII* probe identified several transformants carrying a single insertion, and no band was detected in the wild-type strain. When hybridized with the downstream region of *CaGα1*, single DNA fragments of the same size were observed in the transformants, and a smaller DNA band appeared in the wild-type strain. The data indicate that these strains were gene-specific knockout strains, and no nonspecific insertion in the genome was detected (Figure 3). The gene-deleted strains 2 and 23 were used for further analysis. *CaGα1* complementation strains were generated from the *CaGα1-*deleted strain ∆Gα1-23, and strains E2 and E3 were used for further analysis (Figure 3).

### Ethylene did not enhance spore germination or appressorium formation in CaGα1 mutants

**Figure 4:**
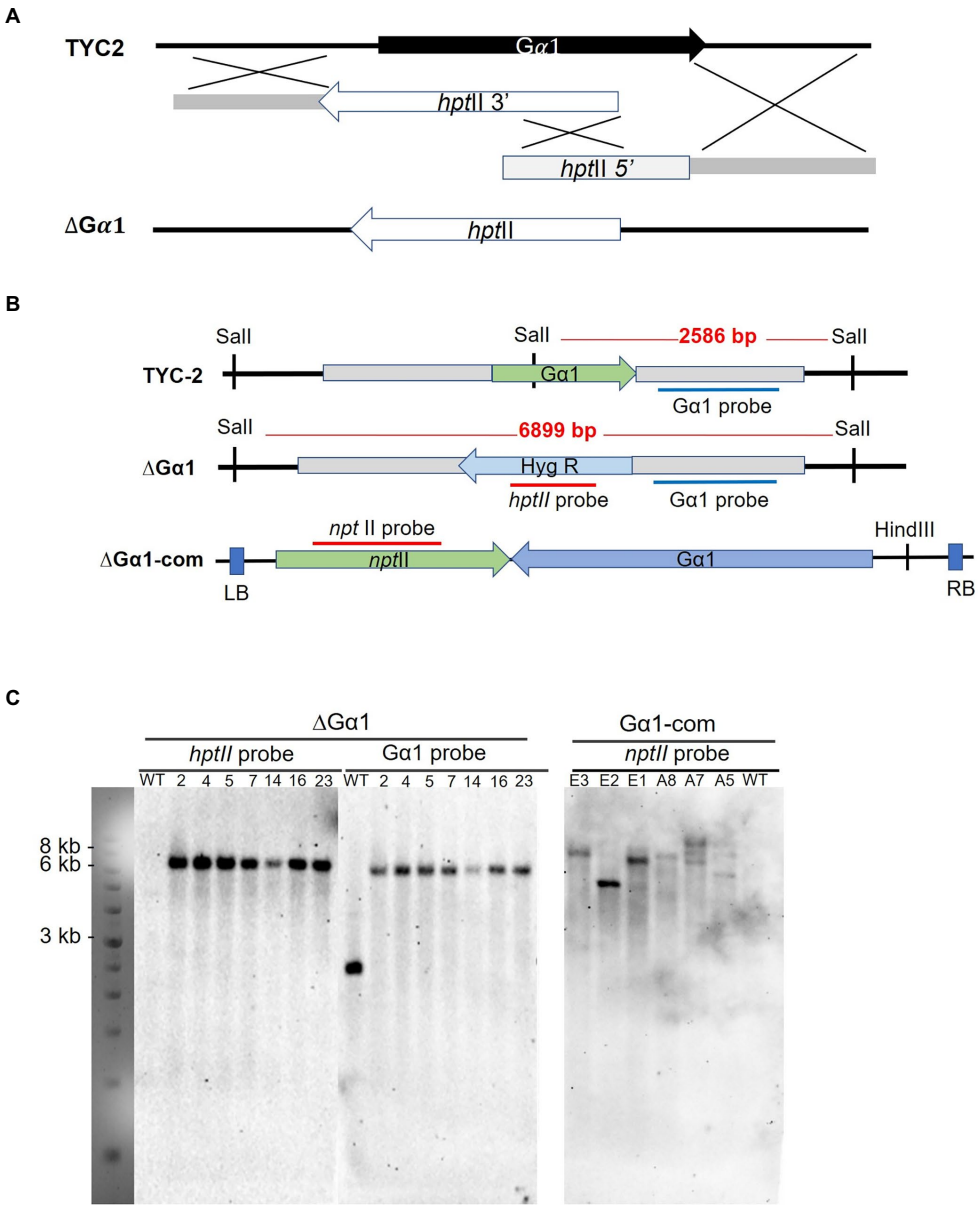
Gene disruption with a split-marker strategy *via* homologous recombination is illustrated **(A)**. The restriction map **(B)** and hybridization results of Southern blot analysis for the CaG𝛼1 gene disruption and complementation strains are presented **(C)**.

Spore germination of the TYC2 and *CaGα1* complementation strains was extremely low under water treatment but significantly stimulated by 1 and 10 μM ethephon. However, stimulation was not found in CaGα1 mutants (Figure 4A), indicating that ethylene-induced spore germination is mediated by CaGα1 signaling in *C. asianum*. Interestingly, spore germination was significantly enhanced in CaGα1 mutants compared to TYC-2 under water treatment on petri dishes. The low germination of TYC-2 in water might be caused by self-inhibition *via* CaGα1 signaling in TYC-2 because germination self-inhibition is often found in *Colletotrichum* species (Leite and Nicholson, 1992; Tsurushima et al., 1995; Inoue et al., 1996).

Appressorium formation was significantly different between CaGα1 mutants and TYC-2 under ethephon treatments (Figures 4A,B). Ethylene enhanced appressorium formation in the TYC-2 and *CaGα1* complementation strains but not in the two mutants. In the water treatment, CaGα1 mutants showed significantly lower appressorium formation than TYC-2 on petri-dish and glass slide. The data suggest that the low appressorium formation of CaGα1 mutants might be due to the loss of response to ethylene or/and other appressorium formation inducers, such as hard surfaces.

### Spore germination inhibition by self-inhibitors was not regulated by CaGα1

**Figure 5:**
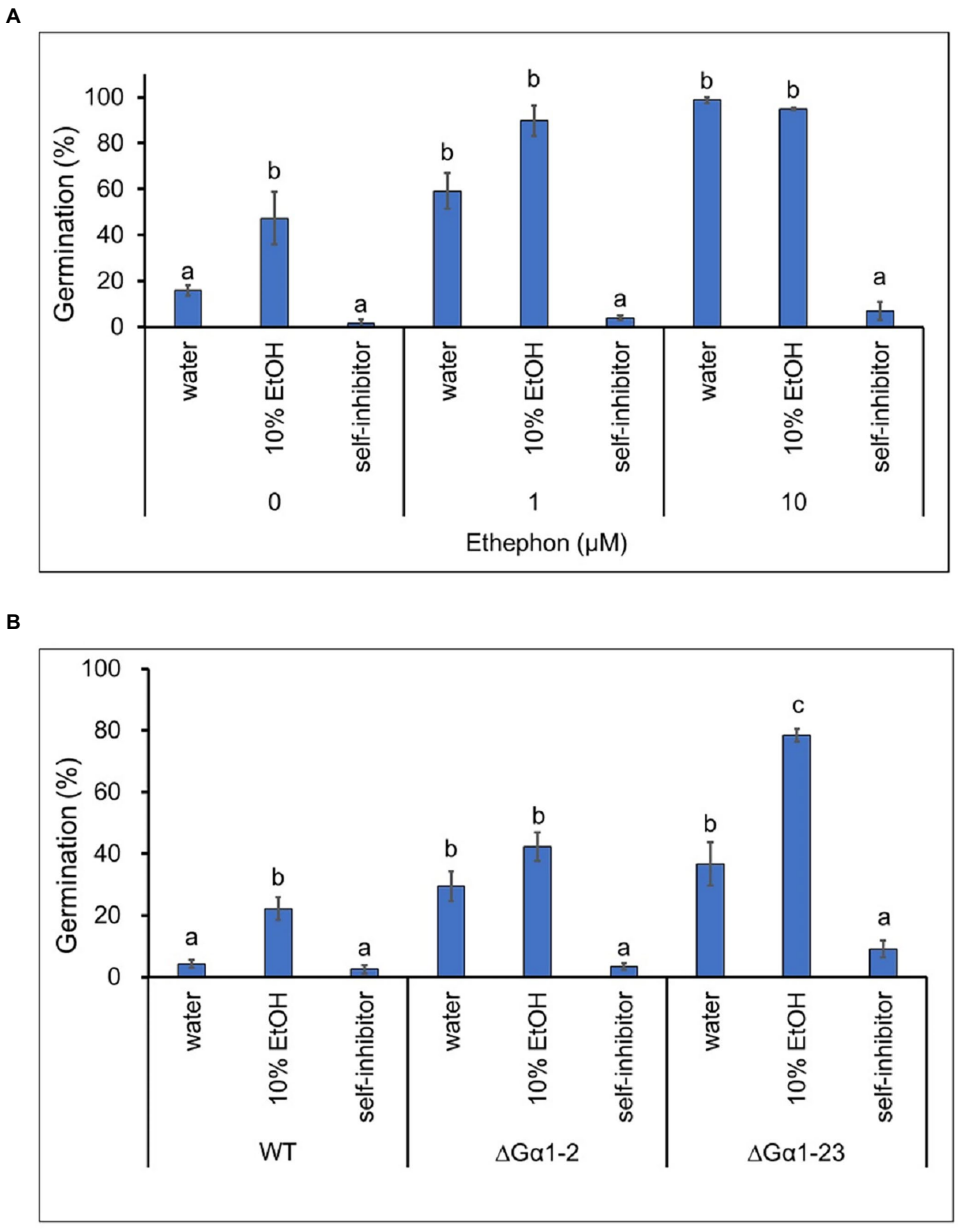
Germination self-inhibition assay of partially purified self-inhibitors on plastic petri dishes at 6 h. **(A)** The effect of self-inhibitors on the germination of *Colletotrichum asianum* wild-type strain (TYC-2) under 0–10 μM ethephon treatment. **(B)** The effect of self-inhibitors on the germination of TYC-2 and G𝛼1 mutants (∆G𝛼1–2, ∆G𝛼1–23) under water treatment. Partially purified self-inhibitors were diluted in 10% ethanol. The data within same treatment were analyzed by one-way ANOVA, different letters indicate significant differences between treatments (*p* value <0.05).

Fungal self-inhibitors are known to be present on spores that prevent germination until the spores are dispersed in a favorable environment (Staples, 1997). Host factors can relieve this self-inhibition (Hedge and Kolattukudy, 1997). Self-inhibitors from *Colletotrichum* species have been well characterized and most of them are small molecules with double bond (Leite and Nicholson, 1992; Tsurushima et al., 1995; Inoue et al., 1996; García-Pajón and Collado, 2003). Self-inhibitors extracted from TYC-2 spores inhibited colony formation on the TLC plate (Supplementary Figure 6). One (fraction A; Rf: 0.19) of the partially purified fractions inhibited spore germination and was used in the following assays. Ethephon significantly enhanced spore germination of TYC-2 in water and in 10% ethanol, but the enhancement was not observed in the self-inhibitor treatment (Figure 5A). This result indicates that ethylene could not overcome the influence of self-inhibitors on spore germination. CaGα1 mutants had a higher spore germination ability in water and 10% ethanol than TYC-2, but the two mutants and TYC-2 strain showed a very low germination ability in the self-inhibitor treatment. This result suggests that spore germination inhibition by this partially purified self-inhibitor fraction is not mediated *via* the CaGα1 signaling pathway (Figure 5B). In addition, the self-inhibition bioassay on the TLC plates from spores of G𝛼1 mutants (∆G𝛼1–2, ∆G𝛼1–23) showed similar self-inhibition ability as the bioassay of TYC-2 at Rf = 0.19 (Supplementary Figure 6A).

### Spore germination of CaGα1 mutants was enhanced on hydrophobic surfaces

**Figure 6:**
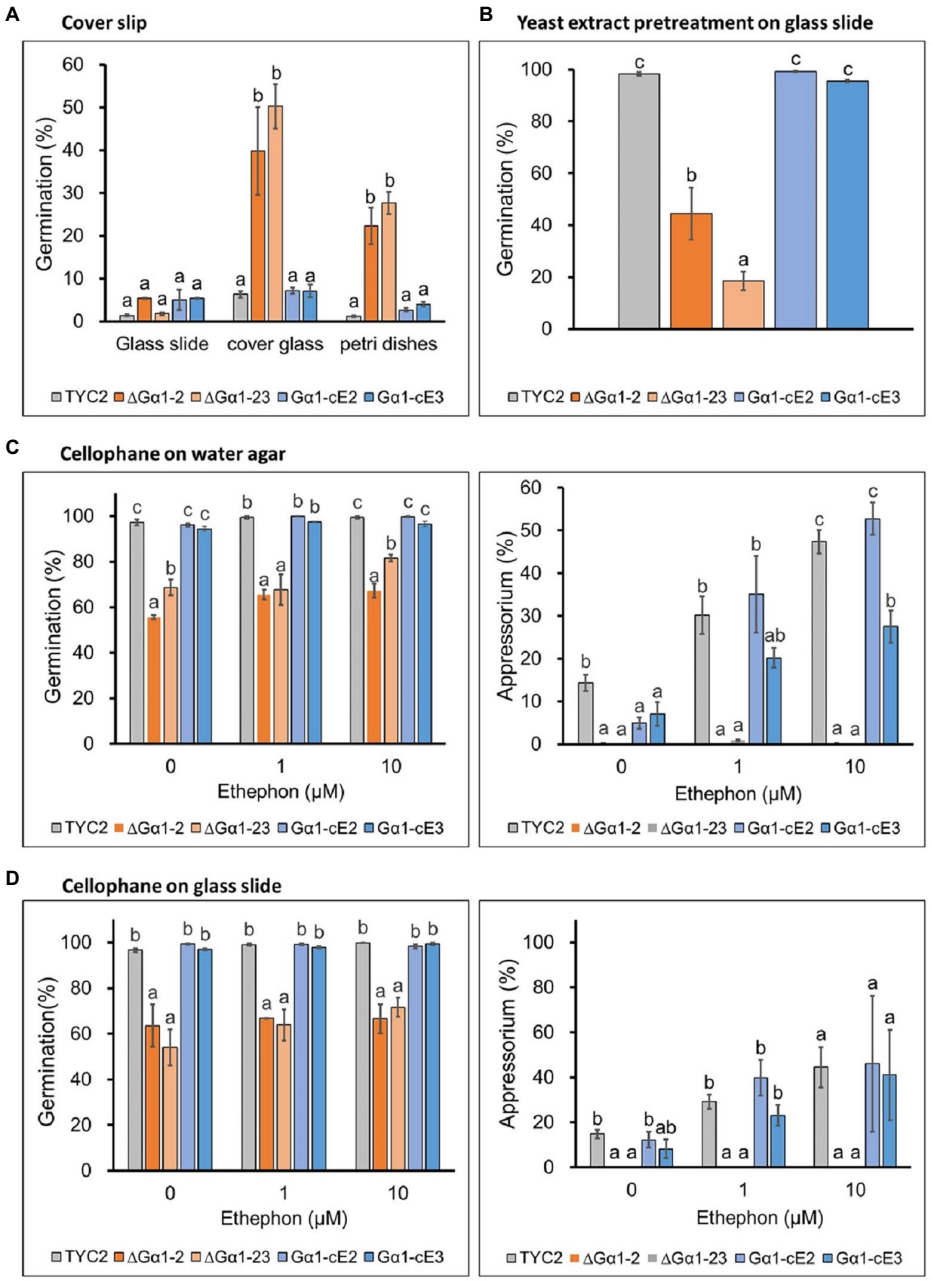
Spore germination and appressorium formation of *Colletotrichum asianum* wild-type strain (TYC-2), G𝛼1 mutants (∆G𝛼1–2, ∆G𝛼1–23) and gene complementation strains (G𝛼1-cE2, G𝛼1-cE3) on different surfaces at 3 h under 0–10 μM ethephon. **(A)** Spore germination on cover slip. **(B)** Spore germination on glass slide after yeast extract pretretment. **(C)** Spore germination and appressorium formation on cellophane topping on 1.5% water agar. **(D)** Spore germination and appressorium formation on cellophane topping on glass slide. The data within same treatment were analyzed by one-way ANOVA, different letters indicate significant differences between treatments (*p* value <0.05).

To understand the influences of different contact surfaces on CaGα1 signaling, glass slides, cover slips, cellophane topping on water and glass slides, and pretreatment with yeast extract on glass slides were tested. In the water treatment, CaGα1 mutants had a higher germination percentage than TYC-2 on the cover slip, the same as that observed on petri dishes (Figures 4A, 6A). The two surfaces are known hydrophobic surfaces (Lee and Dean, 1993). The germination enhancement in CaGα1 mutants in water treatment was not found on glass slides, water agar, cellophane topping on water and glass slides, or yeast extract pretreatment (Figure 6). In ethephon treatments, appressorium formation were enhanced in TYC-2 but not in CaGα1 mutants on all tested surfaces, suggesting that ethylene stimulates appressorium formation of *C. asianum* TYC-2 *via* the mediation of CaGα1 signaling on all the tested contact surfaces. However, a hard surface is required for appressorium formation by *Colletotrichum* species. CaGα1 might be required for appressorium formation when sensing the hard surface but is not involved in ethylene sensing for appressorium formation. In addition, CaGα1 mutants showed significantly lower appressorium formation than TYC-2 on cellophane topping on glass slide and 2% water agar in water treatment. Over 90% germination with no appressorium formation in TYC-2 were observed on cellophane topping on 0.15% water agar (Data not shown). Taken together with the data in water treatment in Figures 4A,B, it indicates that hard surface is required for appressorium formation and CaGα1 mediates hard surface sensing in TYC-2.

Ethylene can induce multiple appressorium formation of TYC-2 (Figure 1). Multiple appressorium formation was significantly enhanced by 10 μM ethephon in the TYC-2 and gene complementation strains but not in the CaGα1 mutants (Figure 4C). This finding indicates that ethylene regulates appressorium formation of TYC-2 *via* CaGα1 signaling.

On cellophane topping on water agar and glass slide and yeast extract pretreatment, strong germination ability was found in TYC-2 in water treatment, suggesting that cellophane might provide nutrients as yeast extract did for TYC-2 germination. However, this germination enhancement was significantly lower in CaGα1 mutants than in TYC-2. These data indicate that germination stimulated by nutrients in TYC-2 is partially mediated *via* the CaGα1 signaling pathway (Figures 6B–D).

### CaGα1 mutants had much less virulence on mango leaf and fruit

**Figure 7:**
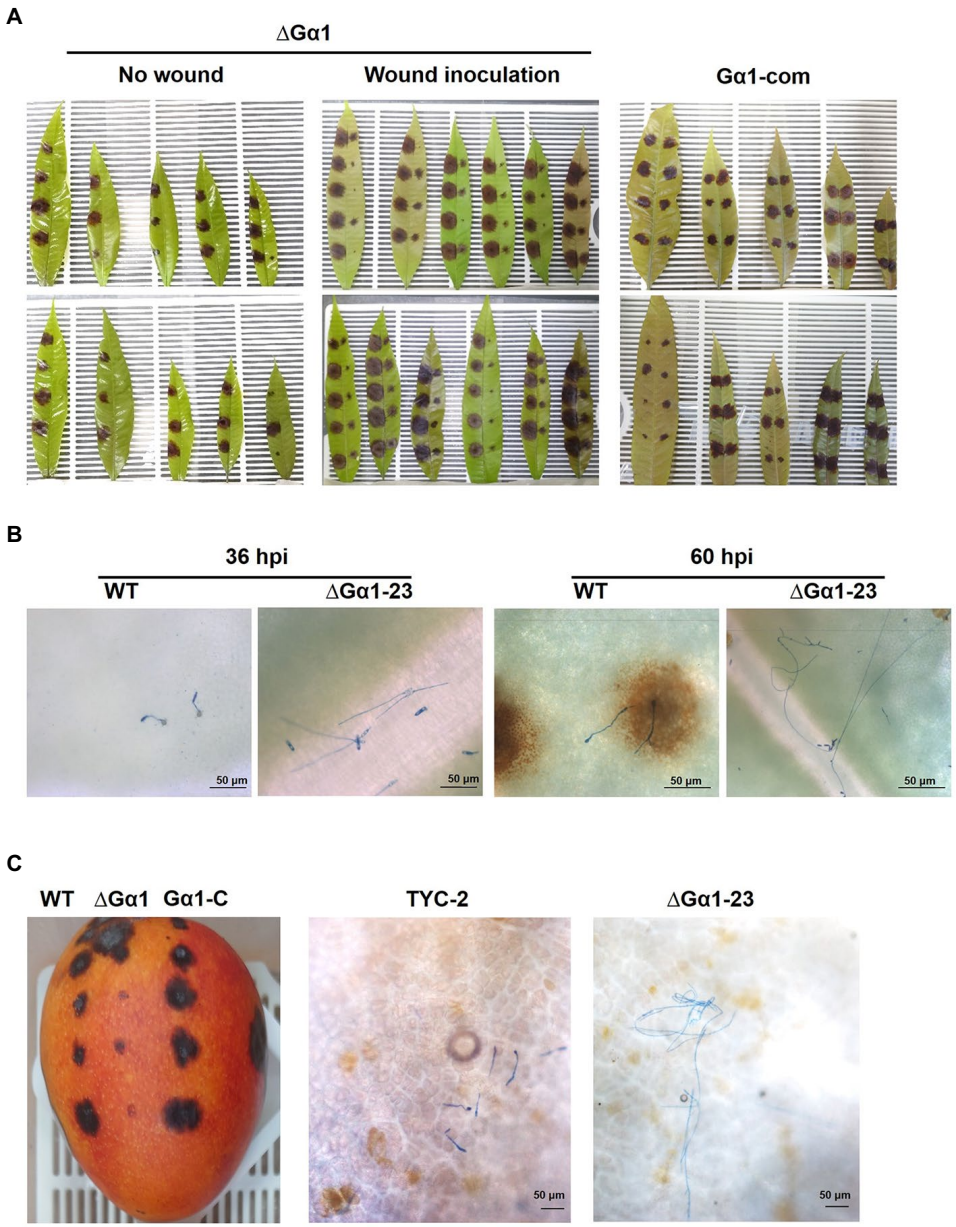
Pathogenicity assay of *Colletotrichum asianum* wild-type strain TYC-2 (WT), G𝛼1 mutants (∆G𝛼1–2, ∆G𝛼1–23) and gene complementation strain (G𝛼1-com) on mango leaf **(A,B)** and fruit **(C)** at 4 and 14 days postinoculation, respectively. On mango leaf, TYC-2 was inoculated on the left side and a mutant or a gene complementation strain was inoculated on the right side of the same leaf **(A)**. Spore germination and hyphal growth on the leaf surface were presented at 36 and 60 h postinoculation (hpi) **(B)** and on the mango fruit surface at 48 hpi **(C)**.

The pathogenicity assay on mango leaf and fruit showed low virulence of CaGα1 mutants (Figure 7). CaGa1 mutants usually did not show symptoms on mango leaves at 4 dpi when TYC2 caused necrotic lesions approximately 1 cm in diameter (Table 1; Figure 7). In the wound inoculation assay, necrotic lesions caused by all strains appeared at 4 dpi, but CaGα1 mutants caused significantly smaller lesions than TYC-2. Pathogenicity assay on mango fruit showed similar results of delayed infection of CaGα1 mutants. The mutants caused smaller lesions than TYC-2 on mango fruits 7 dpi (Figure 7).

**Table 1:** Lesion sizes of mango leaves caused by *Colletotrichum asianum* strains TYC-2 (WT), CaG𝛼1 mutants (∆G𝛼1-2, ∆G𝛼1-23) and gene complementation strains (G𝛼1-cE2, G𝛼1-cE3) at 4 days postinoculation.

Exp.	*N*	Mean lesion area (cm^2^)					Paired *t*-test (*p* values)
		WT	ΔGα1-2	ΔGα1-23	Gα1-cE2	Gα1-cE3	
1	24	0.19	0.002	–	–	–	0.010
	22	0.58	–	0.002	–	–	<0.001
	18	0.95	–	–	0.87	–	0.37
	19	0.53	–	–	–	0.42	0.31
2	15	0.94	0.006	–	–	–	<0.001
	15	1.01	–	0.004	–	–	<0.001
	17	0.47	–	–	0.60	–	0.14
	17	0.85	–	–	–	0.81	0.40
Wound-1	24	2.34	0.70	–	–	–	<0.001
	24	1.64	–	0.36	–	–	<0.001
Wound-2	25	1.92	0.30	–	–	–	<0.001
	27	2.35	–	0.49	–	–	<0.001

N, number of total inoculated spots for mango leaves.

By microscopic examination of the inoculation spot, TYC-2 spores germinated and formed appressoria at 24–36 hpi, but ∆CaGα1-23 spores remained ungerminated at 24 hpi and germinated with long germ tubes at 36 hpi. Single spores forming multiple appressoria were observed in TYC-2 at 48 hpi, but no appressorium formation was found in ∆Gα1-23. Infection and necrotic lesion formation were clearly observed at the infection site of TYC-2 at 60 hpi. In contrast, ∆Gα1-23 grew into long and branched hyphae and did not form appressoria on the mango leaf surface, and no necrotic lesions were observed under a microscope. At 84 hpi, the CaGα1 mutant colonized the infection site surface with abundant hyphae and formed fewer appressoria to penetrate and produce microscopic necrotic lesions. The CaGα1 mutants showed delayed infection of the host. Similar events were found on mango fruits. TYC-2 formed appressoria at 48 hpi, in which the mutants grew into long hyphae without appressorium formation on the fruit surface (Figure 7). This result indicates that the reduced appressorium formation ability and the expression of some virulence genes during infection may be involved in the extremely low virulence of CaGα1 mutants.

### CaGα1 mutants showed smaller colony sizes and produced fewer spores than TYC-2

CaGα1 mutants showed significantly smaller colony sizes on three tested media, rich media PDA and MS, and modified minimal medium Czapek (mCz). CaGα1 mutants produced less orange pigment on MS medium, indicating that their sporulation ability might be affected (Supplementary Figure 7). By quantifying spores from the same colony size, CaGα1 mutants were obviously less sporulation than TYC-2 (Table 2). By measuring the length and width of spores, CaGα1 mutants had slightly longer spores than TYC-2 (Table 2; Supplementary Figure 7C), but the longer spores could germinate and produce appressorium (Supplementary Figure 8).

**Table 2:** Spore size and sporulation of *Colletotrichum asianum* wild-type strain TYC-2 and CaGα1 gene mutants.

Strains	Spore size (μm)*		Sporulation (10^7^ spores/cm^2^)**
	Length	Width	
TYC-2	12.96 ± 1.28 a***	3.53 ± 0.57 a	3.95 ± 0.10 a
ΔGα1-2	14.03 ± 2.30 b	3.61 ± 0.83 a	0.61 ± 0.06 b
ΔGα1-23	14.07 ± 2.59 b	3.30 ± 0.99 a	0.62 ± 0.12 b

*Mean and standard error of spore size, in which three replicates were calculated, at least 100 spores counted each replicate. **Mean and standard error of sporulation calculated with three replicates. Sporulation was presented based on total spores/area of colony. ***The data within same column were analyzed by one-way ANOVA, different letters indicate significant differences between treatments (*p* value < 0.05).

### Virulence-related genes were up- or downregulated by ethylene

**Figure 8:**
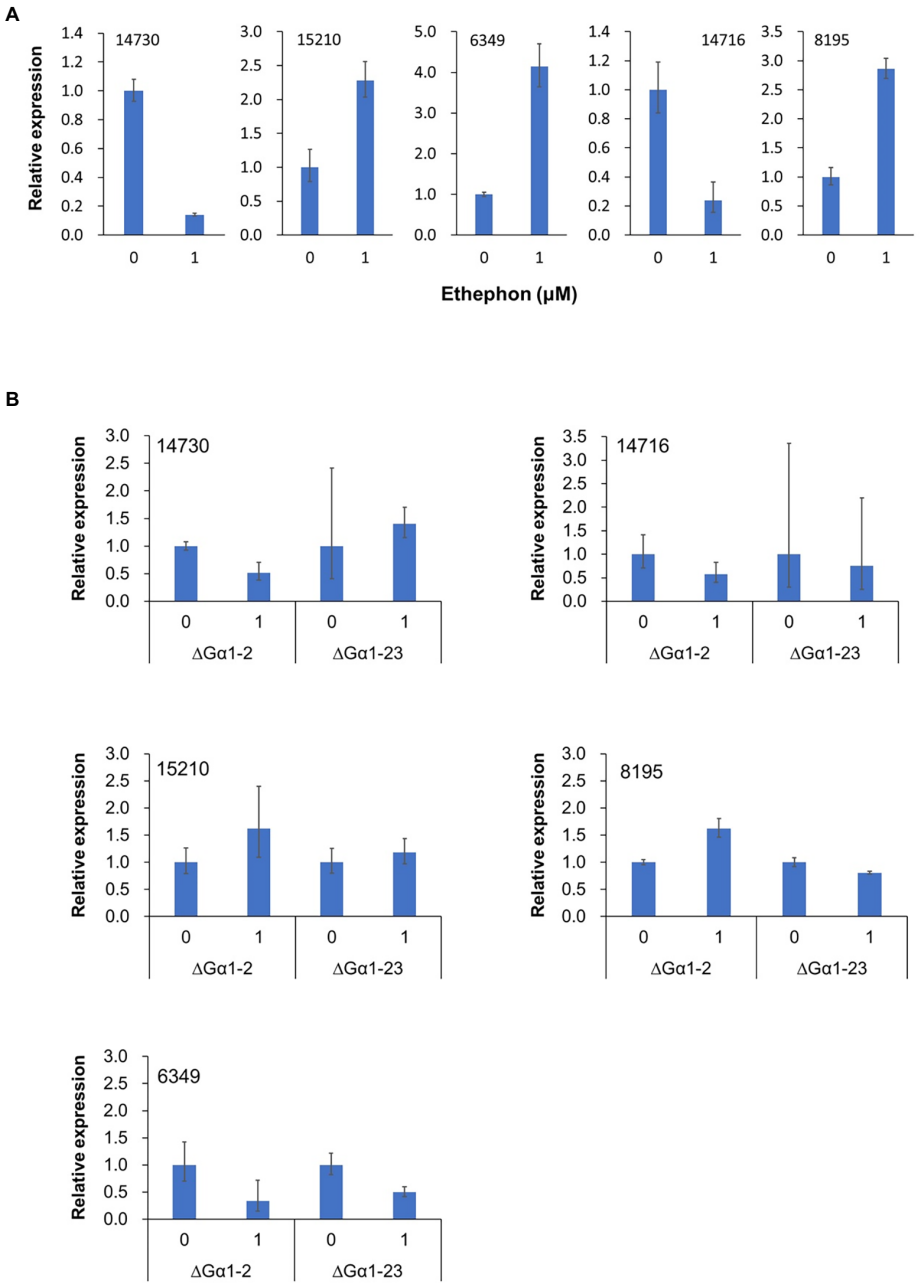
Relative expression of five genes (14,730-hydrophobin, 15,210-cutinase, 14,716-major facilitator family, 8,195-pectate lyase, and 6,349-amino acid permease) in *Colletotrichum asianum* TYC-2 **(A)** and G𝛼1 mutants (∆G𝛼1–2, ∆G𝛼1–23) **(B)** at 3 h after treatment with 0 or 1 μM ethephon. Relative expression was determined using actin gene as internal control and calculated with comparative ΔΔCt method by comparing to water treatment.

Our data clearly demonstrated that TYC-2 can sense ethylene. To identify ethylene-responsive genes in TYC-2, RNA-seq for comparative transcriptomic analysis was performed. Mycelia treated with ethephon were used for RNA-seq. Spores during germination were not used here because we do not want to identify the differentially expressed genes (DEGs) that contain a lot of genes specific for germination and appressorium formation that could be also induced by other factors, such as hard surface, and then interfere the ethylene sensing assays in the future. Fifteen DEGs with *p value*s < 0.05 were identified (Table 3). Three of the 15 genes contained signal peptides, and three genes contained transmembrane domains. Three genes encoding virulence-related proteins, cutinase (15210), pectate lyase (8195), and hydrophobin (14730), and two genes with transmembrane domains (6,349 and 14,716) were analyzed using qRT–PCR for their expression under 1 μM ethephon treatment (Figure 8A). Genes encoding hydrophobin and a major facilitator family protein were consistently downregulated by ethylene, while genes encoding amino acid permease and cutinase were consistently upregulated by ethylene. The gene encoding pectate lyase regulated by ethylene was not highly consistent, with upregulation in three independent experiments and downregulation in one experiment. Furthermore, the expression of the five genes was not regulated by ethylene in the two CaGα1 mutants (Figure 8B). Taken together, the five genes were demonstrated as ethylene responsive genes. When the expression levels of the five genes were compared to TYC-2, the five genes were significantly downregulated in the two CaGα1 mutants in the water and 1 μM ethephon treatments (Supplementary Figure 9), except that pectate lyase was not consistently downregulated in the two mutants in the water treatment, in which pectate lyase was downregulated in the mutant ΔGα1-2 and ΔGα1-23 in two and one, respectively, of the two independent experiments. The data suggest that other external signals might also be involved in the expression of the five genes *via* the mediation of CaGα1.

**Table 3:** List of DEGs (*p* < 0.05) under 1 μM ethephon treatment.

ID	Length (nt)	Sequence desc./pfam	Log2-FC	Value of *p*
SignalP				
**15210** ^a^	669	Cutinase/ pfam 01083.22	−2.52	0.004
**8195**	765	Pectate lyase E/ pfam 03211.13	−1.37	0.042
**14730**	312	Hydrophobin/ pfam 06766.11	−1.59	0.050
TMM				
**6349**	1,395	Amino acid permease (8-TM)/pfam 13520.6	6.59	0.041
15864	1,317	Sugar transporter (9-TM)/pfam00083	1.92	0.031
**14716**	1,623	Major facilitator family (10-TM)/pfam 07690.16	1.86	0.045
Others				
3336	1,638	Ferulic acid decarboxylase 1/pfam 01977.16	2.36	0.0008
3321	834	UbiX family flavin prenyltransferase/pfam 02441.19 (Flavoprotein)	2.38	0.002
8,955	1,746	FAD-dependent monooxygenase/pfam 07992, 13,738, 00743	2.03	0.003
6368	3,864	Glycosyl hydrolases family 35/pfam01301	2.62	0.004
8960	867	Enoyl-(Acyl carrier protein) reductase/pfam13561	1.88	0.007
15861	825	Domain of unknown function (DUF3328)/pfam11807	1.89	0.014
412	759	RCR superfamily; Chitin synthesis regulation/pfam12273	1.71	0.020
3337	2,151	Zn(2)-C6 fungal-type DNA-binding domain protein/Non available	1.45	0.037
11,832	1,308	NAD dependent epimerase/dehydratase family/pfam01370	−1.73	0.027

^a^Bolded gene names were used for qRT-PCR analysis.

## Discussion

Mango anthracnose pathogen *C. asianum* causes severe economic losses in mango fruit production at the preharvest and postharvest stage in Taiwan. *Colletotrichum* infection of climacteric fruits is known to be related to the host ripening hormone ethylene. The sensing and response of the mango pathogen *C. asianum* to ethylene have not been investigated, and the sensing and signaling in *Colletotrichum* species to ethylene remain to be clarified. In this study, we investigate the roles of histidine kinase-containing proteins, similar to plant ethylene receptors, and a G-protein in the ethylene sensing of *C. asianum*. Our results indicate that the G protein subunit α1, CaGα1, mediates ethylene sensing of the mango anthracnose pathogen *C. asianum* to regulate fungal development and virulence and mediates surface sensing for spore germination.

Ethylene is a gas and is not easy to handle. Ethephon can release ethylene at high pH and is commonly used instead of ethylene gas in postharvest treatments and related research (Flaishman and Kolattukudy, 1994; Awad, 2007; Catín et al., 2007). In previous studies regarding *Colletotrichum* species responding to ethylene, ethephon solution was mixed with fungal spores to assay germination and appressorium formation (Kim et al., 2000). In our system, ethephon was not directly in contact with fungal spores or plant tissues, and the ethylene release patterns showed a linear correlation with the incubation time within 6 h of incubation. Ethylene release gradually increases, and stimulation of TYC-2 may not be as fast as treatment with the same concentration of ethylene gas, but it is close to the ethylene release pattern of mango fruit during maturation (Ketsa et al., 1999).

Ethylene can enhance postharvest disease development, either by changing physiological activities in the fruits and/or affecting the growth and development of pathogens (El-Kazzaz et al., 1983; Flaishman and Kolattukudy, 1994; Saltveit, 1999; Sane et al., 2005; Prusky et al., 2013). For example, the application of 1-methycyclopropene, which blocks ethylene perception by plant receptors, inhibits disease development in many diseases, such as tomato fruit to *Alternaria alternata* and mango fruit to *C. gloeosporioides* (Su and Gubler, 2012; Xu et al., 2017). Ethylene can enhance fungal growth and/or development in *Rhizopus stolonifera, B. cinerea* and *Colletotrichum* species (El-Kazzaz et al., 1983; Flaishman and Kolattukudy, 1994; Chagué et al., 2006). In this study, we demonstrated that ethylene released from ethephon can induce spore germination, appressorium formation and lesion size expansion in the mango pathogen *C. asianum*. The larger lesion size in the ethylene treatment than in the control is primarily caused by earlier germination and multiple appressorium formation. However, fungal virulence factors or host responses to ethylene cannot be excluded from a role in the lesion expansion, for example, genes upregulated by ethylene in this study, such as genes encoding cutinase, pectate lyase and amino acid permease.

**Figure 9:**
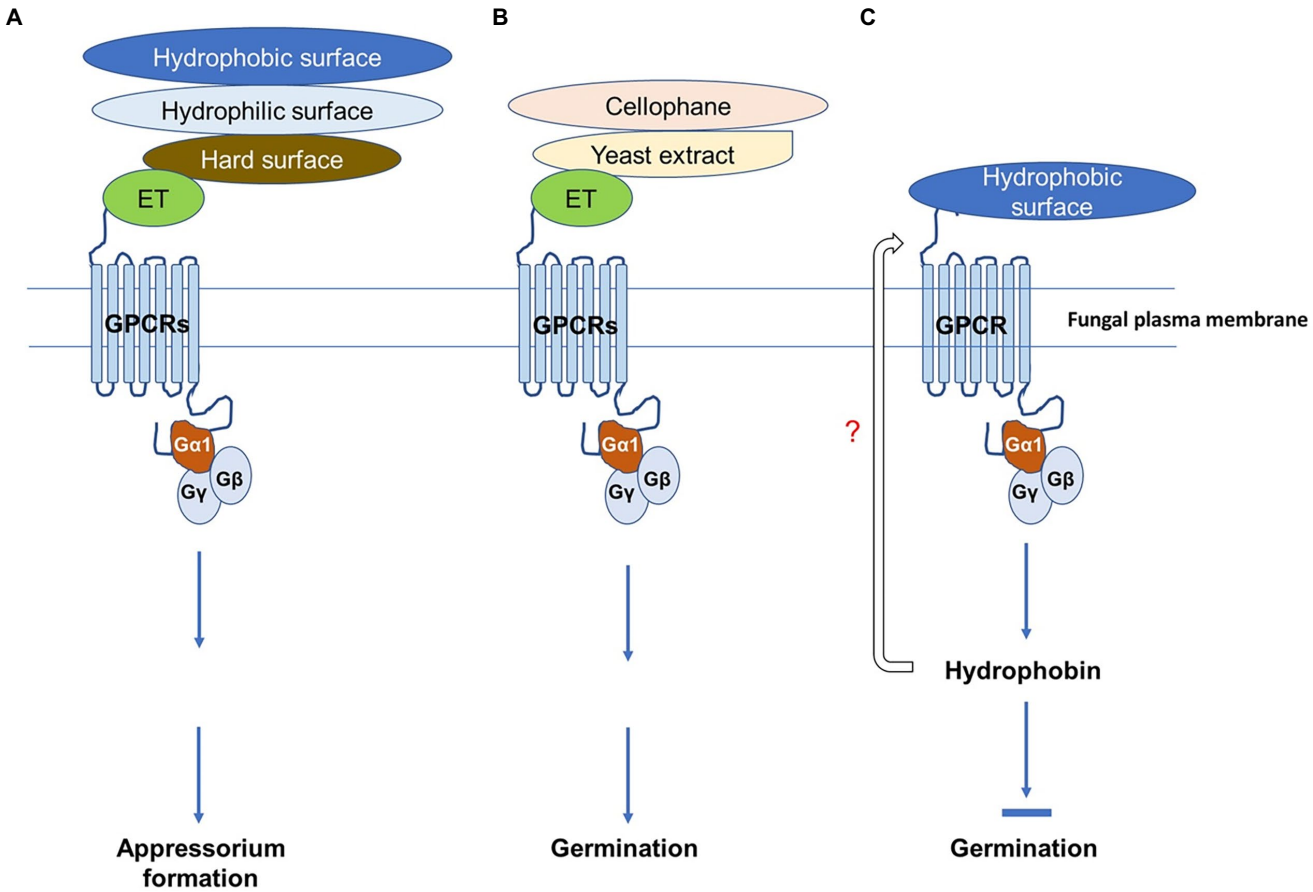
Schematic diagram of spore germination and appressorium formation regulated by different surface cues mediated by CaGα1 (Gα1) signaling in *Colletotrichum asianum* TYC-2. **(A)** Surface cue sensing of *C. asianum* to hydrophobic, hydrophilic and hard surface and the fruit-ripening hormone ethylene for appressorium formation is mediated *via* the G-protein coupled receptor (GPCR)-CaGα1 signaling. **(B)** Chemical sensing of *C. asianum* to cellophane, yeast extract and ethylene for spore germination is mediated *via* the GPCR-CaGα1 signaling. **(C)** Hydrophobin is probably involved in the negative regulation of GPCR-CaGα1 signaling on spore germination of *C. asianum* on hydrophobic surface. In this diagram, different stimuli might bind to different GPCRs, but all signaling pathways shown in this figure are mediated by CaGα1 for spore germination and appressorium formation.

Responses to various environmental cues for spore germination and appressorium formation vary among different *Colletotrichum* species (Podila et al., 1993; Flaishman and Kolattukudy, 1994; Warwar and Dickman, 1996; Chaky et al., 2001). Several signaling pathways are involved, but most of them are involved in the response to hard surfaces (Kim et al., 1998, 2000; Takano et al., 2000; Barhoom and Sharon, 2004; Yamauchi et al., 2004). In this study, five *in vitro* surfaces were assayed, and we found that TYC-2 has a very low germination ability on glass slides, cover slips, and plastic petri dishes but germinates well on cellophane topping on water agar and glass slides and pretreatment with yeast extract. *C. graminicola* has obviously higher germination ability on plastic petri dishes than on glass slides (Chaky et al., 2001), but there is no significant difference for *C. asianum* TYC-2 on the two surfaces. Interestingly, the germination of CaGα1 mutants is enhanced compared to the wild-type on hydrophobic surfaces, cover slips and petri dishes, but the enhancement was not found on glass slides and cellophane topping on water agar and glass slides, which are hydrophilic surfaces (Lee and Dean, 1993). This finding indicates that the hydrophobic surface regulates spore germination of TYC-2 *via* CaGα1 signaling. Fungal hydrophobins play roles in the communication of the fungal surface to the contact surface. Hydrophobins are amphiphilic and highly surface-active proteins that modulate fungal surface properties and make them more likely to immobilize on hydrophobic surfaces (Vasselli and Shaw, 2022). Hydrophobins are involved in spore germination of *Trichoderma guizhouense* (Cai et al., 2021) but have no role in *Botrytis cinerea* spore germination (Mosbach et al., 2011). The hydrophobin gene 14730 of TYC-2 was downregulated in CaGα1 mutants. Therefore, hydrophobin might play a role in TYC-2 germination on hydrophobic surfaces *via* CaGα1 signaling. When TYC-2 spore senses hydrophobic surface, the expression of hydrophobin gene 14730 might be turn on and then affect the sensing and signaling for spore germination (Figure 9). This hypothesis will be investigated in the future. As shown in Figure 9, G-protein interacts with G-protein coupled receptor (GPCR), a large group of membrane receptors in eukaryotes. When an external stimulus binds to a GPCR, such as hydrophobic surface, the associated G-protein would be activated, like CaGα1, and then triggers cellular response to the stimulus *via* the G-protein-GPCR mediated signaling transduction.

CaGα1 mutants showed significantly lower germination than the wild-type under the cellophane and yeast extract treatments, indicating that CaGα1 might be partially involved in the nutrient signaling for germination of TYC-2. In the avocado pathogen *C. gloeosporioides*, CgMEK1, a MAP kinase kinase, is involved in sensing ethylene but not yeast extract in spore germination (Kim et al., 2000). This avocado pathogen has extremely low germination ability on glass slides. Another *C. gloeosporioides*, the rubber tree pathogen, has a very high germination ability on glass slides, but the germination of its Gα1 mutant is highly suppressed (Li et al., 2021). However, in the Gα1 protein mutant of rice blast pathogen *M. oryzae*, appressorium formation on hydrophobic surface is highly reduced, but spore germination is not affected (Liu and Dean, 1997). These studies indicate that *Colletotrichum* pathogens from same species have diverse germination abilities and Gα1 protein mediating fungal germination also varies in different pathogens. Our data demonstrate that ethylene stimulates TYC-2 germination *via* CaGα1 signaling (Figure 9). However, CaGα1 mediates spore germination in response to ethylene, yeast extract and cellophane may be through the interactions with different GPCRs.

Appressorium formation is mediated *via* CaGα1 in TYC-2 in response to ethylene and different physical surfaces, which is summarized in Figure 9. In *M. oryzae,* class I Gα protein and G-protein coupled receptors Pth11 and WISH are required for appressorium formation on hydrophobic surfaces (Kou and Naqvi, 2016; Sabnam and Barman, 2017), while cyclic AMP-dependent and MAPK signal pathways are involved in the downstream signaling of the G-protein pathway in *M. oryzae* for appressorium formation (Lengeler et al., 2000). The two downstream pathways are also involved in appressorium formation in *Colletotrichum* species in response to hard surfaces (Takano et al., 2000; Barhoom and Sharon, 2004; Yamauchi et al., 2004). Therefore, *C. asianum* TYC-2 sensing ethylene *via* G-protein may also have similar downstream signal pathways to those found in *M. oryzae*. The number of GPCR genes is much more than that of Gα in an organism. For example, there are over 800 individual GPCR genes and 16 Gα subunits found in human. It has been known that numerous distinct receptors can couple to the same Gα protein and that the same receptor can also couple to more than one Gα protein (Brown et al., 2018; Wootten et al., 2018). In Figure 9, the GPCRs used to recognize various surfaces, ethylene and nutrients might be different in *C. asianum*, although they all interact with CaGα1 for spore germination and/or appressorium formation. Identification of ethylene receptor GPCR(s) in *C. asianum* will be performed soon in the future and the ethylene responsive genes identified in this study are tested currently for their potentials as reporter genes in ethylene sensing assay.

Histidine kinase proteins (HKs) are key signaling proteins involved in the perception and transduction of environmental stimuli in prokaryotes and eukaryotes (Hérivaux et al., 2016). In this study, three CaHK genes were upregulated by ethylene, but their role in ethylene sensing was not found. In plants, HKs can serve as ethylene binding receptors (ETRs; Binder, 2008). Plant ETR-like HKs have been identified in lower fungi, but not in other fungi or in *C. asianum* (Papon and Binder, 2019; this study). The HKs in TYC-2 may not function as ETR in plants, but it cannot be excluded that they may have roles in the downstream regulation of some uncharacterized phenomena after ethylene sensing in TYC-2. In addition, functional redundancy might be one of the reasons that we did not detect the phenotype of the mutants with a single CaHK gene mutation in ethylene treatment.

To identify ethylene-responsive genes in TYC-2, transcriptomic analysis was performed in this study. Since more than 4,000 genes are upregulated during spore germination and appressorium formation (Liang et al., 2018; Wang et al., 2018), mycelia treated with ethephon were used in comparative transcriptome and qRT–PCR analyses to identify genes specifically regulated by ethylene. Only a few genes were differentially expressed in ethylene treatments in two independent RNA-seq. This is probably because no detectable phenotypes appeared in the ethephon treatment on TYC-2 mycelia. Ethylene-induced transcriptional changes have been studied in *B. cinerea,* and approximately 61 genes have been identified as putative ethylene-responsive genes (Chagué et al., 2006). Four of these genes are pathogenicity-related genes (cyanide hydratase, endopolygalacturonase 1, trichodiene oxygenase P450, and snodprot 1), but they were not differentially expressed in this study. Hydrophobin, cutinase, and petecte lyase are involved in fungal pathogenicity (Beckerman and Ebbole, 1996; Miyara et al., 2008; Lee et al., 2010). Therefore, the low virulence of CaGα1 mutants may in part be contributed by the ethylene-responsive genes identified here. Cutinase and pectate lyase are known virulence factors of *Colletotrichum* species (Dickman and Patil, 1986; Miyara et al., 2008). Hydophobin is a virulence factor in *M. oryzae*. Therefore, hydrophobins of TYC-2 are highly suspected to be involved in the germination enhancement of CaGα1 mutants on hydrophobic surfaces. Further investigation of the function of hydrophobins in TYC-2 development and virulence will be conducted. Intriguingly, all five genes assayed by qRT-PCR are downregulated in CaGα1 mutants when compared to the wild-type. It is possible that CaGα1 interacts not only with the ethylene-GPCR but also with other external stimuli-GPCRs in the RNA-seq assay system, such as sensing the nutrients in PDA and then mediating the expression of pectate lyase, cutinase and amino permease.

In conclusion, this study demonstrates that spore germination, appressorium formation and virulence of the mango pathogen *C. asianum* are regulated by ethylene *via* the signaling of CaGa1. In addition, we discovered that CaGa1 is a negative regulator of hydrophobic surface sensing for spore germination. Several virulence-related genes have been identified as ethylene-responsive genes in *C. asianum*.

## Data availability statement

The original contributions presented in the study are publicly available. This data can be found at: https://www.ncbi.nlm.nih.gov/sra/PRJNA892226.

## Author contributions

M-HL, C-TW, C-YK, and H-CL contributed to the design of the experiments. C-YK performed gene functional analyses of CaGα1, CaHKMp and CaHKGp, and transcriptome analysis as well as qPCR analyses of the selected genes. H-CL performed gene functional analyses of CaHK2 and qPCR analysis of the 12 HK genes. D-KH contributed to TYC-2 genome sequencing and bioinformatic analysis, while H-LL conducted the ethylene detection. M-HL and C-TW supervised the experiments. C-YK, M-HL, and C-TW wrote the manuscript. All authors contributed to the article and approved the submitted version.

## Funding

This research was funded by the Ministry of Science and Technology in Taiwan (grant number MOST 107-2313-B-005-032-MY3 and MOST 110-2313-B-005-013-MY3), and the Advanced Plant Biotechnology Center from the Featured Areas Research Center Program within the framework of the Higher Education Sprout Project by the Ministry of Education in Taiwan.

## Conflict of interest

The authors declare that the research was conducted in the absence of any commercial or financial relationships that could be construed as a potential conflict of interest.

## Publisher’s note

All claims expressed in this article are solely those of the authors and do not necessarily represent those of their affiliated organizations, or those of the publisher, the editors and the reviewers. Any product that may be evaluated in this article, or claim that may be made by its manufacturer, is not guaranteed or endorsed by the publisher.

## Footnotes

^1^ https://www.ncbi.nlm.nih.gov/tools/primer-blast/
