# Supplementary material for: The G protein subunit α1, CaGα1, mediates ethylene sensing of mango anthracnose pathogen Colletotrichum asianum to regulate fungal development and virulence and mediates surface sensing for spore germination
Source: Front Microbiol. 2022 Nov 24;13:1048447. doi: 10.3389/fmicb.2022.1048447 (PMC9731116; doi:10.3389/fmicb.2022.1048447)
Supplement: Supplementary file 1 [file Data_Sheet_1.PDF]

Supplementary Table 1. The primers used in qRT-PCR assay.

**primer name**	**Sequence (5'→ 3')**	**Accession number**
Actin-qPCR-F	ATCAACCCCAAGTCCAACAG	OP142727
Actin-qPCR-R	GGCGTTGAAGGTCTCGAAG	
8195-qPCR-F	CGAGGATGCTCTCTCCCTGA	OP142732
8195-qPCR-R	TGTGCTGGATGACCTTGTCC	
15210-qPCR-F	TCCTTGGAAACATCGCCACC	OP142729
15210-qPCR-R	CAGCGGCTCCTTGGAGTAGC	
14716-qPCR-F	AAGCTGCGTCGGAAGTTGTA	OP142731
14716-qPCR-R	ACCCCAGTGTTGACTTGTCG	
14730-qPCR-F	TTCAGAAAGATCTGCGCGGT	OP142728
14730-qPCR-R	CAGGGGTTTGGCAAAGAACG	
6349-qPCR-F	GTGGTCAGTCTTCGGGGTATT	OP142730
6349-qPCR-R	GTCCTGGAAACTGGCCATCTA	
CaGα1-qPCR-F	CAACCGTATGCAGGAAGCCT	ON936863
CaGα1-qPCR-R	GGCAACTTCTCCTTGAAACGG	
CaHK1-qPCR-F	GGTCTTCAGCCTGTCGATTC	ON936851
CaHK1-qPCR-R	ATAGCGCACCAGAGCTTGAC	
CaHK2-qPCR-F	TTTTCAAAAGCAGCGAACG	ON936852
CaHK2-qPCR-R	CAAGAATAGCACGCTCTCGAC	
CaHK3-qPCR-F	GGTTCAGCGCTACCTCCA	ON936853
CaHK3-qPCR-R	GTGGCGACGTTGAGAGAAG	
CaHK4-qPCR-F	AAAGTGCAGCAGTTCTTGACC	ON936854
CaHK4-qPCR-R	TCCAATCTTCCACTCCTTGAG	
CaHK5-qPCR-F	CCTCGTTTGACGGAAACC	ON936855
CaHK5-qPCR-R	AGACTTTCGGATCACGTTGG	
CaHK6-qPCT-F	ATGACAACACCATGCACAGC	ON936856
CaHK6-qPCR-R	GTTCTCGAGGGCAGGAATC	
CaHK2p-qPCR-F	CGAAAAAGTTCGCGTCTCTC	ON936857
CaHK2p-qPCR-R	GGATTGGTCTGGAGCGTATT	
CaHK5p-qPCR-F	CCGCCAAAACCCATACAG	ON936858
CaHK5p-qPCR-R	ACGTCGGGGAAGGCATAC	
CaHK6p-qPCR-F	ACAGCGGTTTCAACTTCGAT	ON936859
CaHK6p-qPCR-R	CATCGCTTCGGTAGGAACC	
CaHK13p-qPCR-F	GAAGAGAAGAAGCGGTGGAA	ON936860
CaHK13p-qPCR-R	GGCTGGTGATGTCGATGAA	
CaHKGp-qPCR-F	CACAGGCYACCCCGACAG	ON936862
CaHKGp-qPCR-R	CCTTCAAGGACATAAGAGAATGC	
CaHKMp-qPCR-F	CACGTGATCGACGAGTCTTCT	ON936861
CaHKMp-qPCR-R	CTCCGTCGCAAGAACAAGAT	

Supplementary Table 2. The primers used to generate and confirm the transgenic strains in this study.

Primer name	Sequences (5'→ 3')	Purpose
HygR3	GGATGCCTCCGCTCGAAGTA	Homologous recombination of hptII (829 bp)
HygR5	CTTAAGTTCGCCTTCCTCC	
Cg-G-alpha1-3'-R-XbaI	ggttctagaTGCTCTCGGGACTAGCCTTA	Amplification of 3’ flanking of Gα1 (1048 bp)
Cg-G-alpha1-3'-F-SacI	ggtgagctcACCACGGAGCTGGTACAAAG	
Cg-G-alpha1-5'-R-SacI	ggtgagctcCGGCGGATAGAAAAGAATTG	Amplification of 5’ flanking of Gα1 (1046 bp)
Cg-G-alpha1-5'-F-XbaI	ggttctagaGGTAAAGGAGGACGTTGCTG	
Cg-HK2-ko-5’-F-XbaI	ggtctagaCCGATCAGCTGGCATTTGCGAAAT	Amplification of 5’ flanking of CgHK2 (1012 bp)
Cg-HK2-ko-5’-R-SacI	ggtgagcrcCTTTCTGATATCGACGCGTGAC	
Cg-HK2-ko-3’-F-SacI	ggtgagctcCATCATCATGAACCTGCTGG	Amplification of 3’ flanking of CgHK2 (1060 bp)
Cg-HK2-ko-3’-R-XbaI	ggtctagaTCCGGAGAGAACACGTCGTTT	
Cg-HKGp-ko-5’-F-XbaI	ggttctagaGTACCTGCGTCTAAAGGAGACA	Amplification of 5’ flanking of CgHKGp (1076 bp)
Cg-HKGp-ko-5’-R-SacI	ggtgagctcTTTAAGCTCTCGGTCAGGATCG	
Cg-HKGp-ko-3’-F-SacI	ggtgagctcCAATCTACTCACCAACGCCCTC	Amplification of 3’ flanking of CgHKGp (1078 bp)
Cg-HKGp-ko-3’-R-XbaI	ggttctagaCTTCTCAATCAGCTCCGGCATC	
CgHKMp –ko-5’-F-XbaI	ggttctagaTTCGCCCTTCCTGAAAGCCT	Amplification of 5’ flanking of CgHKMp (1070 bp)
CgHKMp –ko-5’-R-SacI	ggtgagctcGAGGTAGTTACTCGCTTGGG	
CgHKMp –ko-3’-F-SacI	ggtgagctcCGACACTGCGAGCACCAGAA	Amplification of 3’ flanking of CgHKGp (1036 bp)
CgHKMp –ko-3’-R-XbaI	ggttctagaTCGGCGTCTTCTCTGTATGG	
G-alpha1-5'-ck-F	TGGACGGCACATTACGAATA	Confirmation of homologous recombination of 5’ flanking
CgHK2-ck-F	GCCGTCTACAACGATGTCAACA	
CgHKGp-ck-F	ACCTACCTAAAATGGTCCCTCGTC	
CgHKMp-ck-F	GGCATGTTCTTCCTCGTCTC	
Hygck-R	GACCGATGGCTGTGTAGAAGTA	
G-alpha1-3'-ck-R	GCATGTCCAACGTCAAAATG	Confirmation of homologous recombination of 3’ flanking
CgHK2-ck-R	TACGAGGCCAGGATCTTGAGGTT	
CgHKGp-ck-R	TTATAGTACGGGCGACGCTG	
CgHKMp-ck-R	TACTTCTGGCCCCAAATGCG	
HygR6	GGCTGATCTGACCAGTTGCCTA	

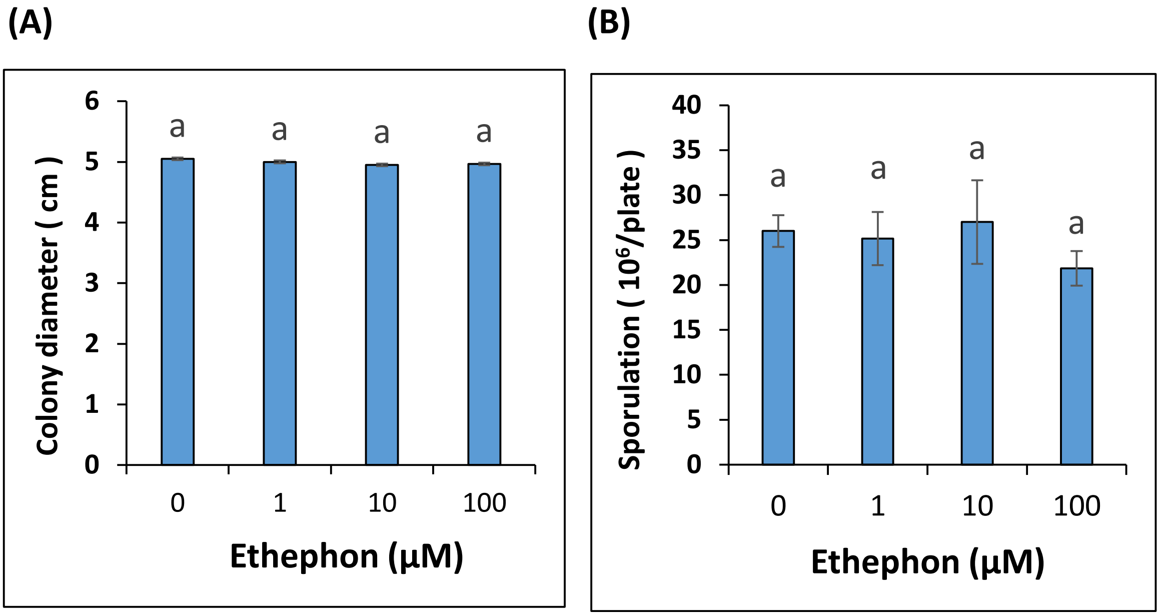

Supplementary Figure 1. Fungal growth (A) and sporulation (B) of *Colletotrichum asianum* TYC-2 under 0-100 µM ethephon treatment. (A). Colony diameter of TYC-2 growing on PDA medium at 4 days post-inoculation. (B). sporulation of TYC-2 after growing on MS agar medium for 4 days. The data were analyzed by one-way ANOVA, different letters indicate significant differences between treatments (p value < 0.05).

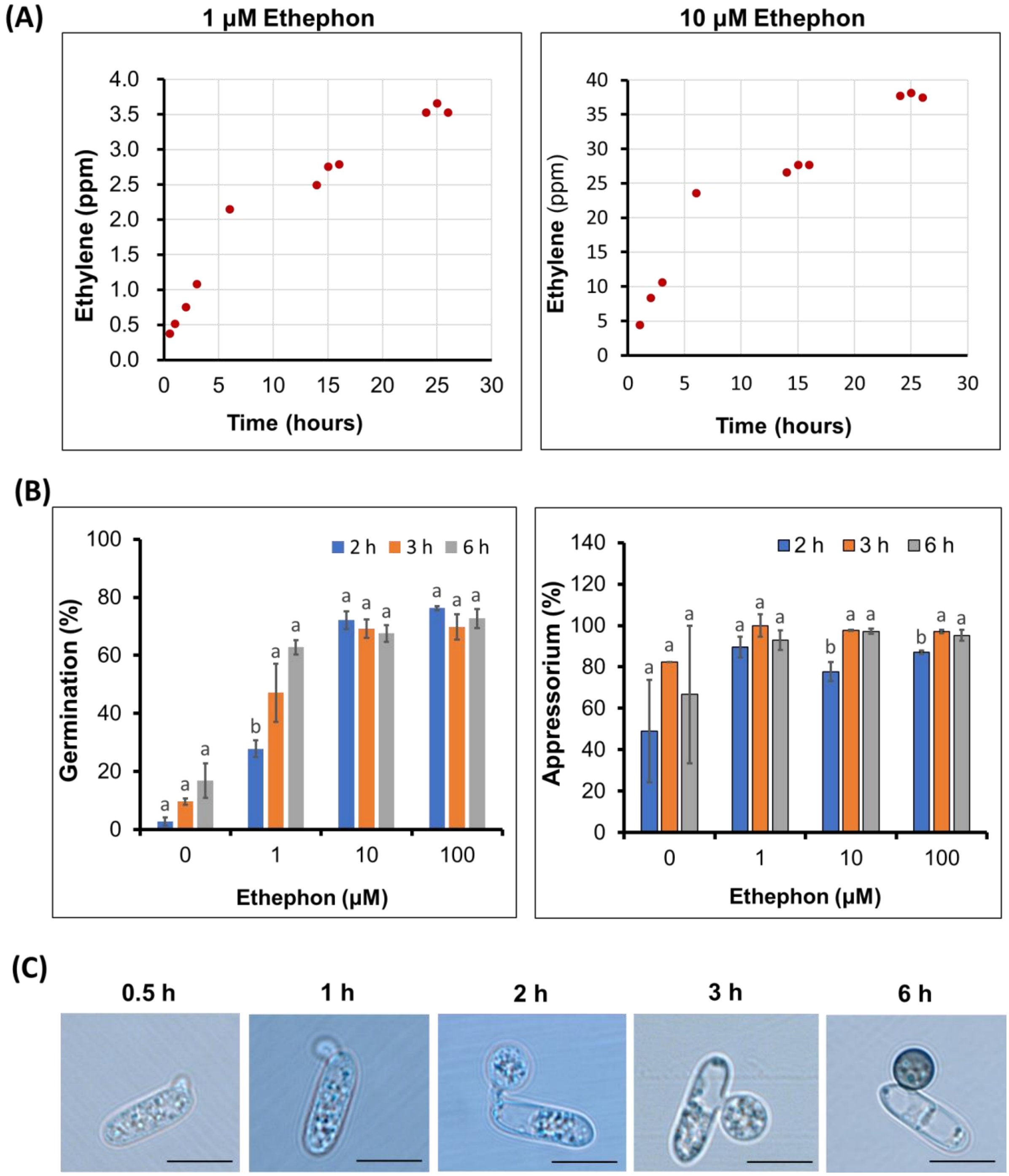

Supplementary Figure 2. Ethylene released from 1 and 10 μM ethephon solution after incubation for 1-26 h (A) and the effects of ethylene on spore germination and appressorium formation (B-C) of *Colletotrichum asianum* TYC-2. (A). The concentration of ethylene released from the ethephon solution at each time point. (B). Spore germination and appressorium formation of TYC-2 under 0-100 μM ethphon treatments at 2, 3, and 6 h postincubation. (C). Spore germination and appressorium formation processes under 10 μM ethephon treatment. Scale bars represent 10 μm.

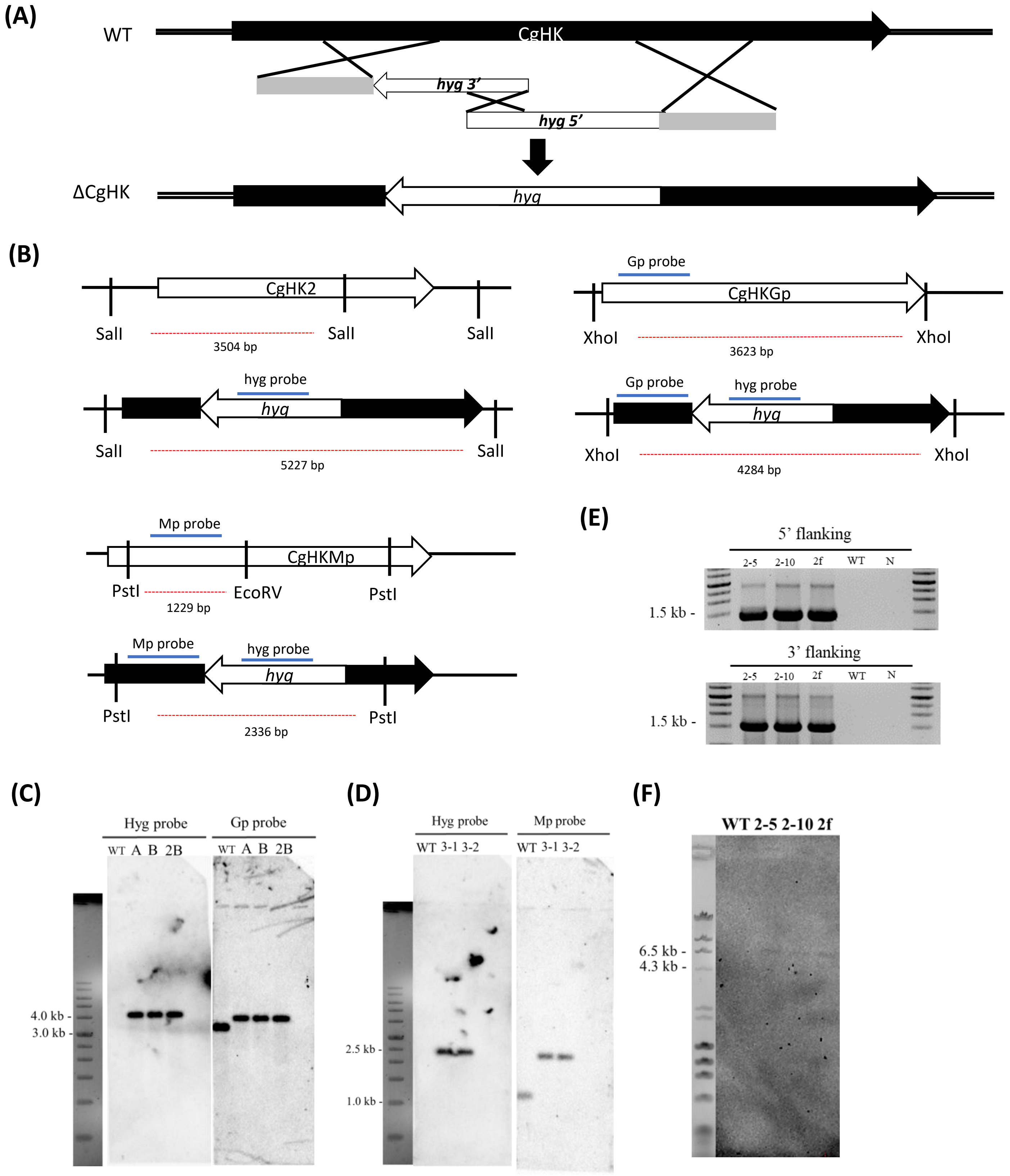

Supplementary Figure 3. PCR screening and Southern blotting of gene disrupted mutants of CaHK2, CaHKMp and CaHKGp of *Colletotrichum asianum* TYC-2. (A). Schematic diagram of gene replacement with split markers. (B). Restriction maps for Southern blotting in wild-type and mutants. (C, D, and F). Southern blotting images of CaHKGp mutants (C), CaHKMp mutants (D), and CaHK2 mutants (F, hybridized with hptII (Hyg) probe). (E). PCR screening for the crossover fragments of 5' or 3' flanking regions (CaHK2 was used to represent this experiment).

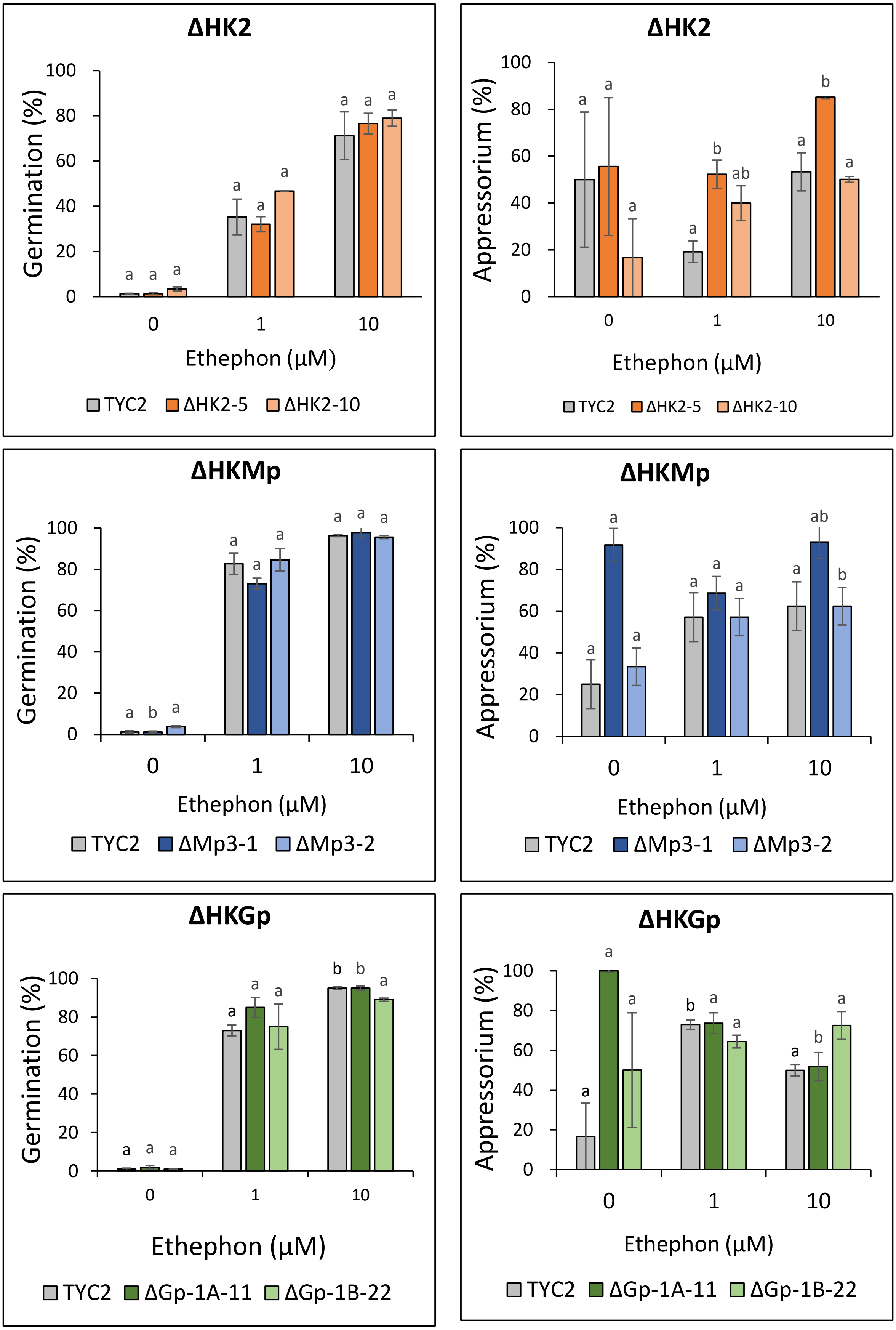

Supplementary Figure S4. Spore germination and appressorium formation of the gene disrupted mutants of CaHK2, CgHKMp and CaHKGp on petri-dish at 3 h under 0-10 μM ethephon treatments. The data within same treatment were analyzed by one-way ANOVA, different letters indicate significant differences between treatments (p value < 0.05).

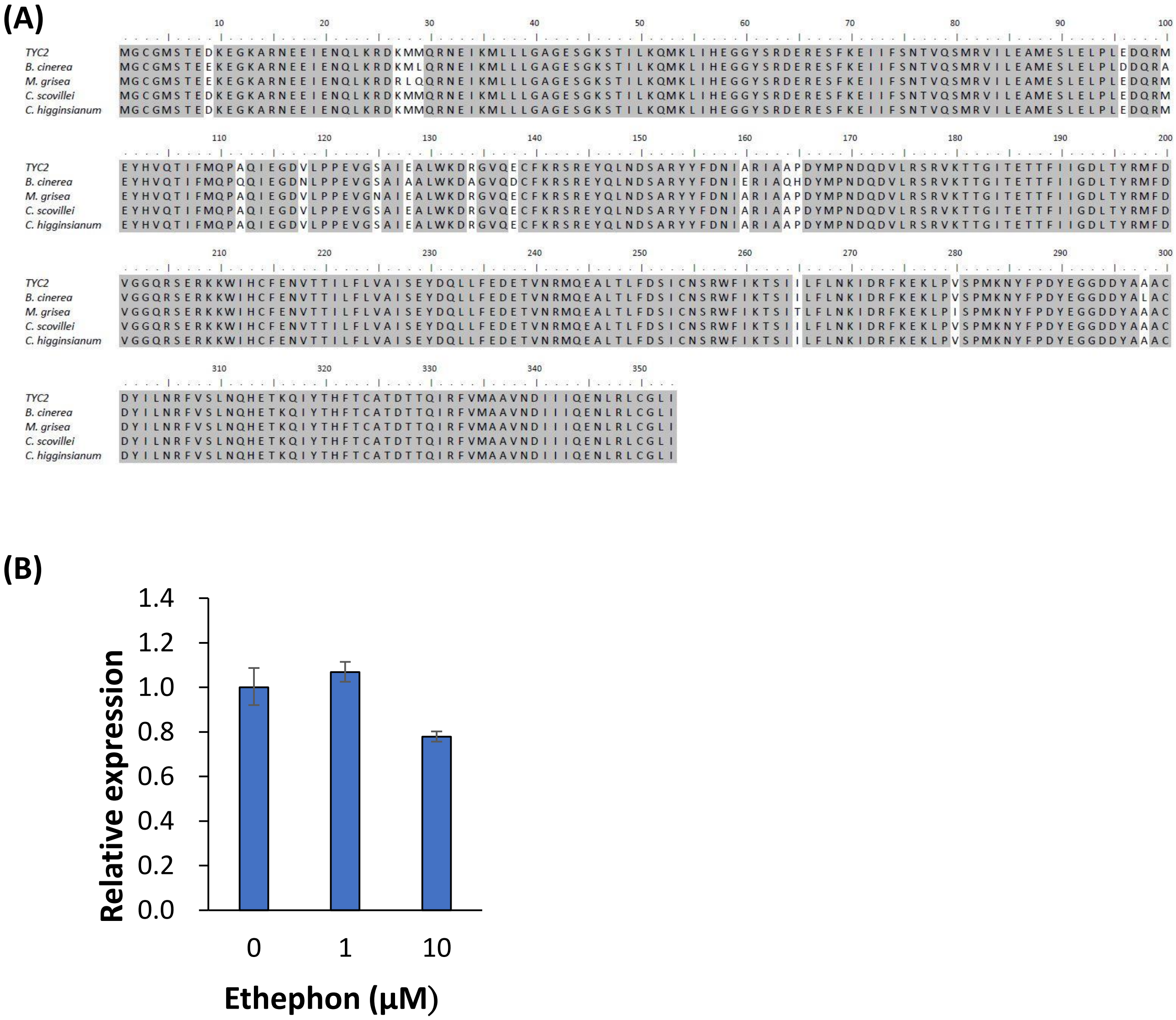

Supplementary Figure 5. Amino acid sequence alignments of Class I Gα1 protein from 4 fungal species (A) and gene expression of CaGα1 under ethephon treatments (B). (A). Amino acid sequence alignments of CaGα1 with the homologs from other fungal species including, *Botrytis cinerea* B05.10 (CAC19871.1), *Magnaporthe grisea 70-15* (AAB65426. 1), *Colletotrichum scovillei 524* (KAG7056029.1) and *Colletotrichum higginsianum* IMI349063 (XP_018162181.1). (B). Relative expression levels of CaGα1 at 3 h under ethephon treatment.

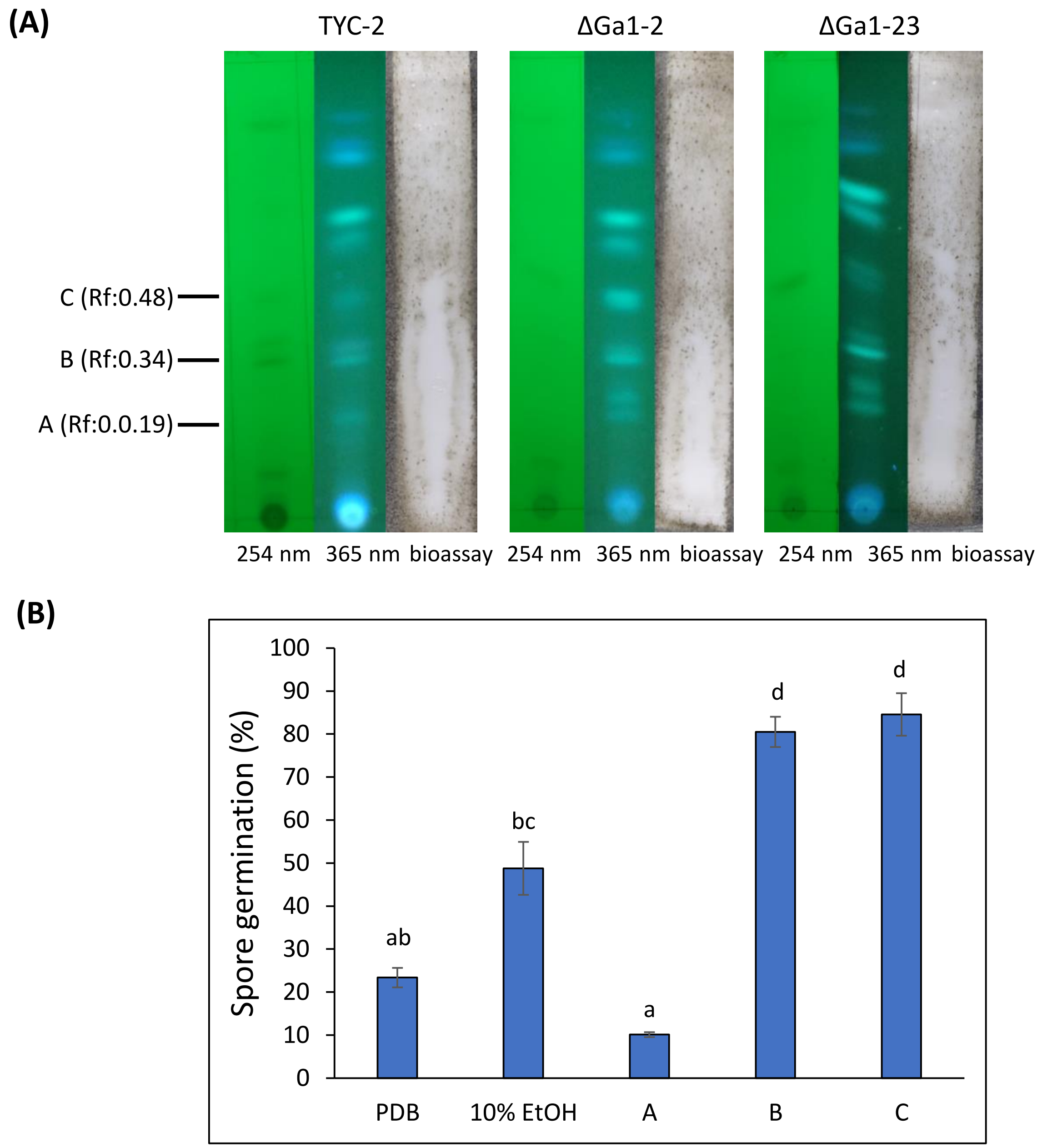

Supplementary Figure 6. TLC-bioassay of self-inhibitors isolated from TYC-2 spores (left panel in A) and their activities on spore germination of TYC-2 (B). Self-inhibitors were separated by TLC and assayed with overplayed with TYC-2 spore suspension amended in PDA and then stained with cotton blue for clear zones. Three fractions of self-inhibitors were collected at the locations as indicated (A, B and C in the left panel). The collected fractions were dissolved in 10% ethanol for germination assay. The fraction A was used for further assays in Figure 5. The TLC-bioassay of self-inhibitors isolated from the spores of Gα1 mutants (ΔGα1-2, ΔGα1-23) were also presented (A). The data were analyzed by one-way ANOVA, different letters indicate significant differences between treatments (p value < 0.05).

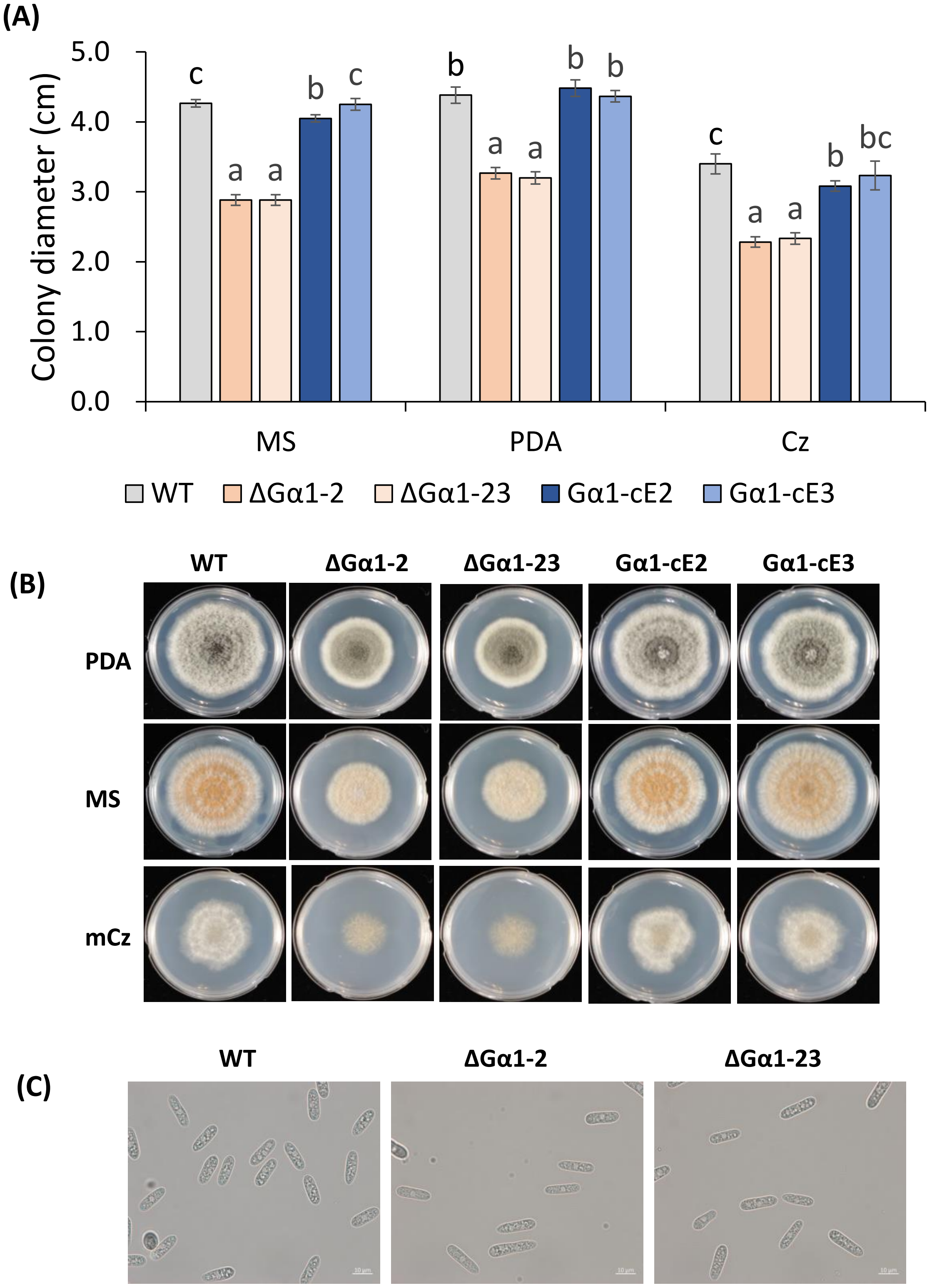

Supplementary Figure 7. Growth of *Colletotrichum asianum* TYC-2 (WT), Gα1 mutants (, ΔGα1-2, ΔGα1-23) and gene complementation strains (Gα1-cE2, Gα1-cE3) on PDA, MS and modified Czapek-Dox (mCz) at 6 days postinoculation (A, B). The data within same treatment were analyzed by one-way ANOVA, different letters indicate significant differences between treatments (p value < 0.05). (C) Spore morphology of TYC-2, ΔGα1-2 and ΔGα1-23 examined under 1000X magnification.

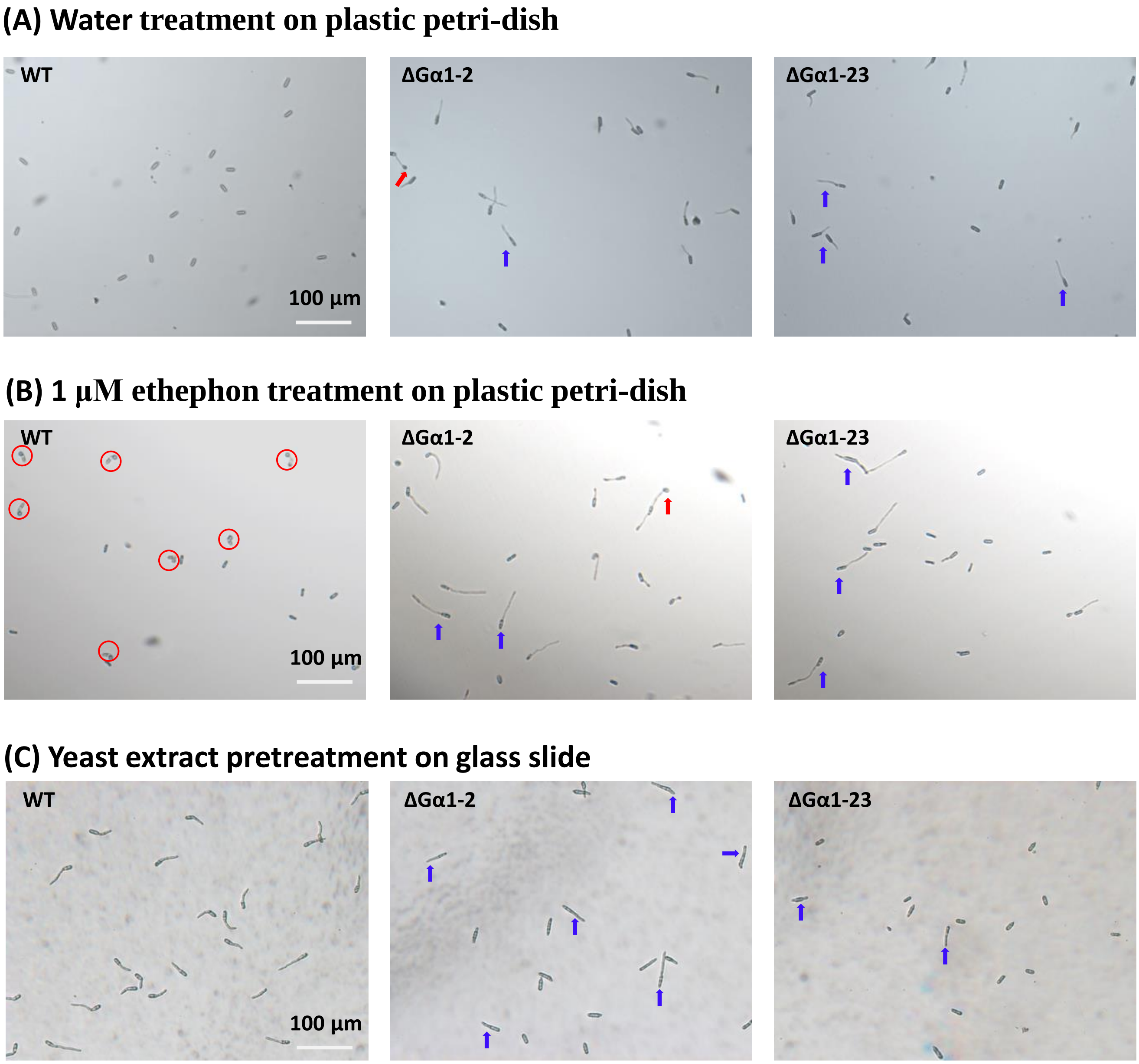

Supplementary Figure 8. Spore germination and appressorium formation of spores of *Colletotrichum asianum* TYC-2 (WT), Gα1 mutants (ΔGα1-2, ΔGα1-23) under different treatments at 3 (A, B) and 6 h post-incubation. The germination and appressorium formation were examined under 100X magnification. Some of long spores of the mutants with germ tube (blue arrows) or appressorium (red arrows) were indicated. Appressorium formation in TYC-2 was marked with red circles.

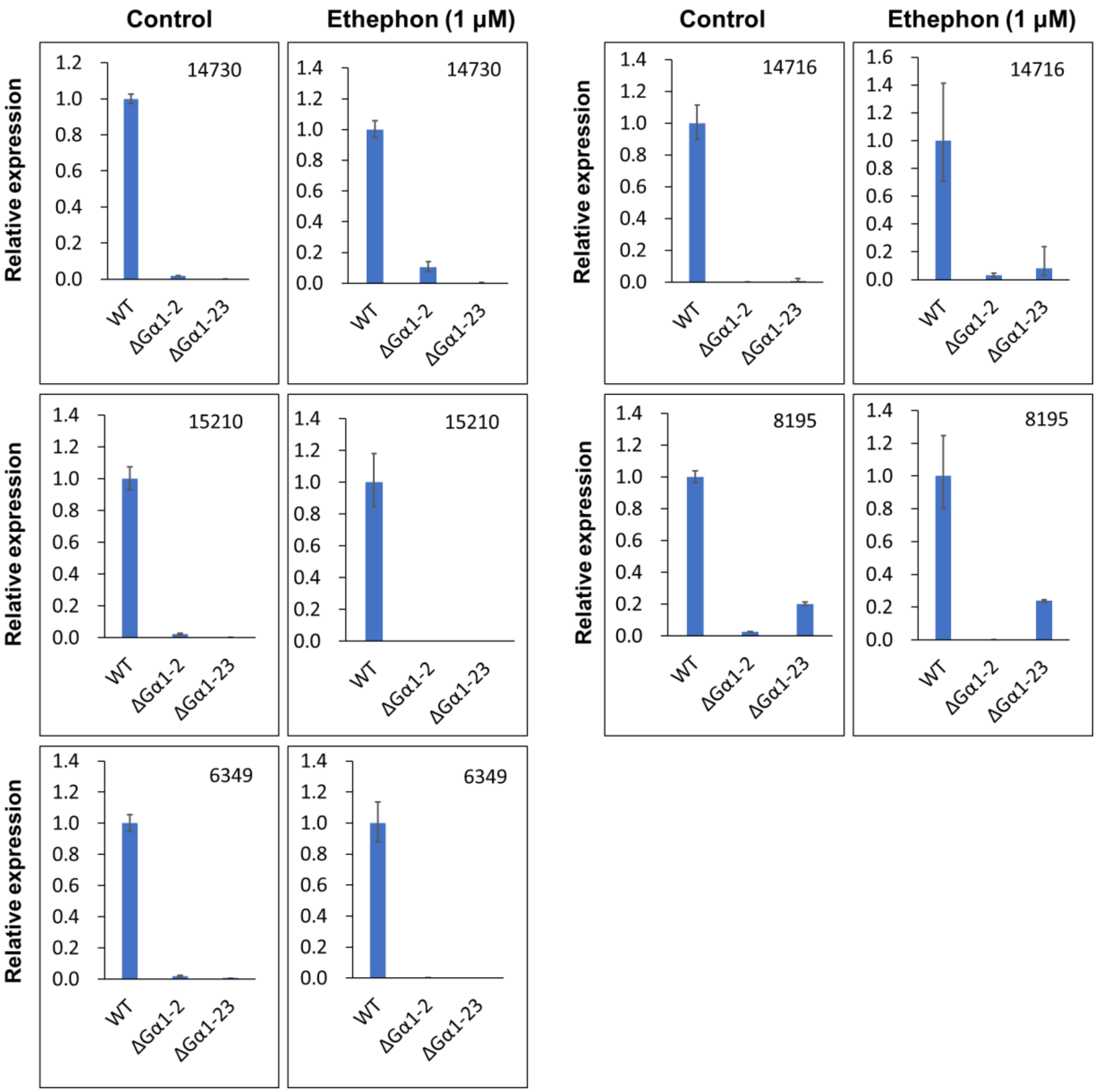

Supplementary Figure 9. Relative expression of five genes (14730-hydrophobin, 15210-cutinase, 14716-major facilitator family, 8195-pectate lyase, and 6349-amino acid permease) in *Colletotrichum asianum* TYC-2 (WT) and Gα1 mutants (ΔGα1-2, ΔGα1-23) at 3 h after treatment with 0 or 1 µM ethephon. Relative expression was determined using actin gene as control and calculated with comparative ΔΔCt method by comparing to TYC-2 (WT).
